# Ant groups optimally amplify the effect of transiently informed individuals

**DOI:** 10.1038/ncomms8729

**Published:** 2015-07-28

**Authors:** Aviram Gelblum, Itai Pinkoviezky, Ehud Fonio, Abhijit Ghosh, Nir Gov, Ofer Feinerman

**Affiliations:** 1 Department of Physics of Complex Systems, Weizmann Institute of Science, Rehovot 7610001, Israel; 2 Department of Chemical Physics, Weizmann Institute of Science, Rehovot 7610001, Israel

## Abstract

To cooperatively transport a large load, it is important that carriers conform in their efforts and align their forces. A downside of behavioural conformism is that it may decrease the group's responsiveness to external information. Combining experiment and theory, we show how ants optimize collective transport. On the single-ant scale, optimization stems from decision rules that balance individuality and compliance. Macroscopically, these rules poise the system at the transition between random walk and ballistic motion where the collective response to the steering of a single informed ant is maximized. We relate this peak in response to the divergence of susceptibility at a phase transition. Our theoretical models predict that the ant-load system can be transitioned through the critical point of this mesoscopic system by varying its size; we present experiments supporting these predictions. Our findings show that efficient group-level processes can arise from transient amplification of individual-based knowledge.

## Abstract (web summary)

Group conformity is crucial for collective behaviours, but may decrease overall responsiveness to external cues. Here the authors show that load-carrying ant groups function at a transition between ballistic and random motions, where the influence of informed individuals is maximized.

## Graphical abstract

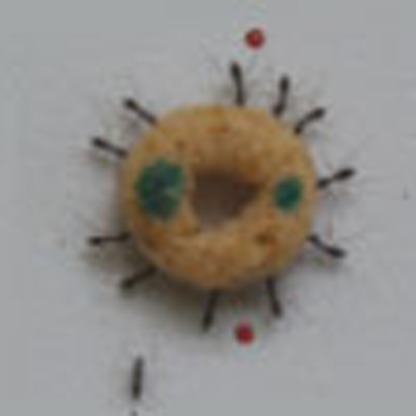

Collectively carrying a large load requires a high degree of coordination and is rare outside humans and ants. One facet of this coordination is the requirement to align forces such that inefficient tug-of-wars are avoided^1^,^2^. Indeed, it is known that group-living animals increase their level of coordination as conformist group members align their actions with those of their neighbours^3^,^4^,^5^. A downside of behavioural conformism is, however, that it may increase the stability of maladaptive behaviours and decrease the group's responsiveness to external cues^6^,^7^,^8^. This problem sharpens if information is scarce or held by a small number of individuals^9^,^10^,^11^,^12^,^13^,^14^,^15^. The advantages and drawbacks of behavioural conformism are accentuated in the case of cooperative load retrieval by ant teams^2^,^16^,^17^,^18^: the large degree of conformity, required to ensure efficient carrying, may hinder the continuous responsiveness needed for navigation through a ragged terrain.

Critical behaviour has been suggested as an organizing principle^19^ in several collective biological systems^20^,^21^. The transition between order and disorder is believed to allow the group to exhibit behaviours that are stable while maintaining flexibility. Cooperative transport by ants provides a unique opportunity to approach these ideas. A main reason for this is that, in this case, the collective goal of the group is simple and well understood: bringing the food to the nest. This allows for a quantitative assessment of the relative contributions of different levels of persistence and responsiveness to the collective goal. Here we combine experiment and theory to study the interplay between conformity and flexibility in groups of *Paratrechina longicornis* ants as they carry a large food item towards their nest^16^.

*P. longicornis* is an invasive species with worldwide distribution that occupies a diversity of habitats from urban to rainforest^22^. These ants have a seasonal preference for proteins, and insects compose a main part of their diet during the summer months^23^. Since these insects are often much larger than the size of worker ants (2.5–3 mm), impressive displays of *P. longicornis* cooperative transport can be observed during this time^23^.

Here we describe field experiments of cooperative transport by *P. longicornis* ants. By combining the analysis of the load motion with single-ant trajectory data, we find that while the combined force of the group determines the speed of the load, it is individual informed ants that steer the direction of movement. To understand how an individual may influence the entire group, we present a microscopically realistic model that is formulated in the language of single-ant decision rules and stands in quantitative agreement with the macroscopic characteristics of the load's motion. The model suggests that carrying ants exhibit an intermediate level of social conformism which, on the collective scale, allows a single individual to optimally steer the load. By mapping our model to a more abstract Ising model, we show how this optimality is related to the divergence of susceptibility at a critical point. The mean-field nature of this model implies that the system can be transitioned through this critical point by varying load size. We provide experimental verification for this prediction.

## Results

### Experimental design and trajectory characteristics

**Figure 1:**
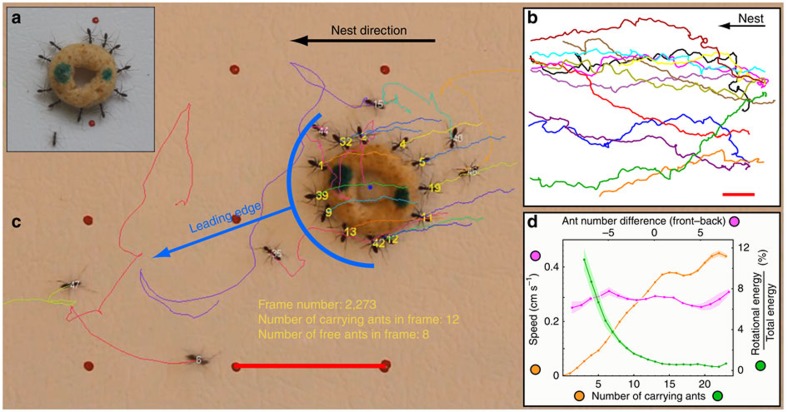
Cooperative transport. (**a**) A team of *P. longicornis* ants retrieving a food item ∼350 times the mass of a single ant. (**b**) Sample nest-bound load trajectories of ∼1-m length each. Scale bar, 10 cm. (**c**) Snapshot from a tracked movie. Carriers are enumerated in yellow and non-carriers in white. The recent trajectories of different ants are marked by different colours. The object's direction of movement is depicted by the blue arrow. Scale bar, 2 cm. (**d**) The natural variability in the number of carriers and their distribution around the load were used to extract median load speed as a function of either total ant number (orange line) or difference in ant occupancy between the leading and the trailing edge (pink line). The green line depicts the efficiency of the carrying in terms of the fraction of energy devoted to rotation. Error bars are the s.d. of a distribution of medians calculated for 1,000 samples bootstrapped from the data. *N*=637,696 Frames.

Ring-shaped food items were placed several metres away from a *P. longicornis* nest in the field. Following discovery of the food and a recruitment phase, the ants commenced cooperative transport (Fig. 1a; Methods; Supplementary Note 1). We repeatedly filmed this behaviour over a distance of ∼1 m (ca. 500 ant lengths) and extracted the positions (Fig. 1b) and angular orientations of the load from a total trajectory length of over 70 m. We also measured the positions of all ants around the object as well as the angles between the carrying ants' body axis and the object radius (Fig. 1c; Methods; Supplementary Movie 1). We further logged all events of ant attachment to and detachment from the load.

Focusing on global features of the load trajectory, we find that while median speed increases linearly with number of carrying ants (up to 15 attached ants), it does not strongly depend on how these ants are distributed around the object (Fig. 1d, orange and pink correspondingly; see Supplementary Note 2). Furthermore, the relative amount of energy wasted on rotating the object rather than translating it decreases with number of carrying ants (Fig. 1d, green). These properties indicate a high level of conformism that enables all attached ants to efficiently align their efforts. This result stands in agreement with cooperative carrying in other ant species^1^,^2^. In contrast, while the trajectories generally head in the correct nest-bound direction they, nevertheless, exhibit substantial sinuosity, looping and detours (Fig. 1b).

### Load steering

Understanding load steering is facilitated by the simple mechanical nature of the system. First, whatever the sources of information may be^24^,^25^, the movement of the rigid load is determined by the sum of forces and torques applied by the carrying ants. Overall forces dictate linear load velocity and angular speed, both of which are experimentally measureable. Second, the forces that ants can exert on the load are constrained by their anatomy: ants can either lift objects or apply pulling forces that are, in general, aligned with their body axis^26^, while pushing is rare to absent^27^ (Supplementary Note 3; Supplementary Fig. 1). Finally, the pulling forces applied by a single ant are of the order of 0.1 mN (Supplementary Note 4).

There are many ways in which a given net force may be distributed between the individual ants. In one extreme case, all ants pull with equal forces and load directionality is set by the angular distribution of attached ants along its edges. However, we find minimal correlation between ant angular distribution and direction of load movement (Pearson Correlation: *r*=0.104, *t*-test: *P*<10^−13^, *N*=5,591 frames from 47 experiments; see Supplementary Note 5 and Supplementary Fig. 2a). This tug-of-war assumption is further incompatible with the linear, rather than square root, rise in the median load velocity with the number of attached ants (Fig. 1d).

At the other end of the spectrum is the case where, although all ants can apply a force, they are actually enslaved by a single-informed ant that guides the entire motion. Indeed, in most of our experiments, we could identify at least one ant that acted as a carrier for the full duration of the retrieval. However, the distribution of correlations between the orientations of these persistently attached ants with the direction of motion of the cargo cannot be distinguished from that of non-persistent ants (*t*-test: *P*=0.6691, *N*_persistent_=14 ants, *N*_non-persistent_=186 ants from five experiments; see Supplementary Note 5 and Supplementary Fig. 2b).

We therefore conclude that the collective movement does not arise neither from a wisdom-of-the-crowds type averaging over all opinions nor by the continuous leadership of any single individual. Next, we aim at examining how the total force applied to the load is distributed among the carrying ants.

### Transient influence of newly attached ants

**Figure 2:**
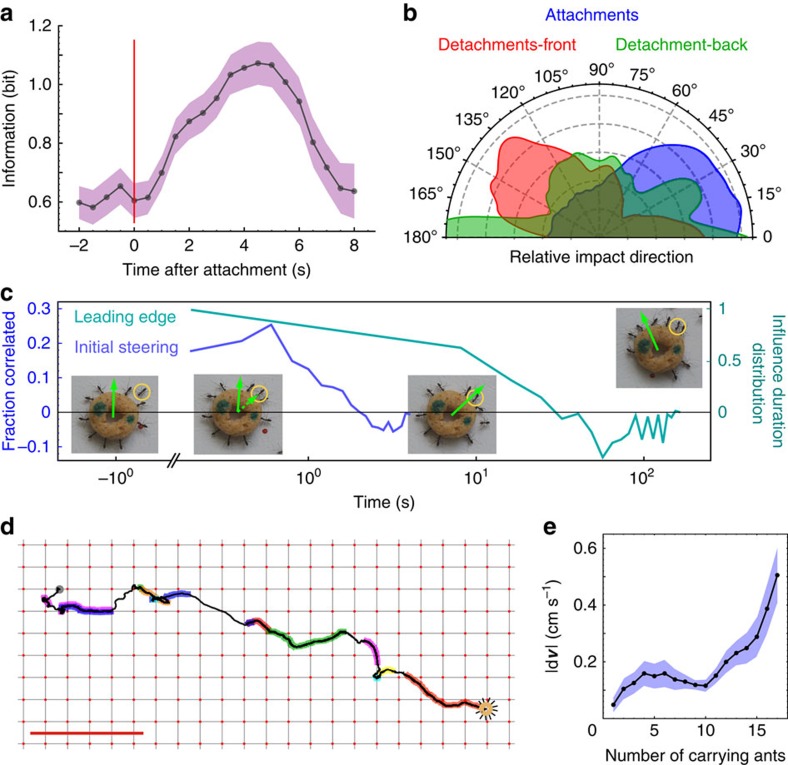
Transient guidance. (**a**) The information in the angular spread of the load's direction of motion immediately following the attachment of a new ant at *t*=0 (*N*=134 attachments). Errors were calculated from the entropy of artificially generated histograms with added binomial noise. (**b**) A half-polar histogram of the angles between the attachment/detachment point of an ant and the change in velocity (relative impact direction) that follows different events (*N*=252). (**c**) Relative impact direction as a function of time (blue line *N*=134 attachments) and difference between distributions of time since attachment of ants in the leading and trailing edge of the load (turquoise line). The insets illustrate this process for a sample newly attached ant (marked by a yellow circle). Load velocity (green arrow) and the acceleration caused by this ant (dashed arrow) are overlaid. (**d**) An example of a series of switches between steering ants along a trajectory. Overlaid colours mark trajectory segments where different ants steered the load. Scale bar, 10 cm. **e**) Mean magnitude of velocity change caused by newly attached ants (denoted by 
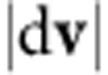
 on *y* axis) as a function of number of ants already attached (*N*=134 attachments). Error bars are standard error of the mean.

Freely moving ants are well informed of the correct nest-bound direction (Supplementary Note 6; Supplementary Fig. 3a). When such ants attach to the load they steer it so that it moves more accurately towards the nest. Quantitatively, we found that within the several seconds that follow their attachment, ants inject about 0.5 bits of directional information into the system (see Fig. 2a, Supplementary Note 6 and Supplementary Fig. 3b). This causal effect implies that newly attached ants adopt an influential role.

The peak in directional accuracy, evident in Fig. 2a, implies that carrying ants are less informed than newly attached ants. The quick deterioration of accuracy after its initial rise shows that while newly attached ants have a transient positive influence, their directional knowledge rapidly decreases following the time of attachment (Fig. 2a). A likely explanation to this effect is obstruction of ants' antennae by the large load (these ants do not strongly rely on vision for their navigation; see Supplementary Note 7 and Supplementary Fig. 4). The timescale of a newly attached ant's influence is much shorter than the entire duration of the transport, which is qualitatively different from more stable forms of influence observed in systems with distinct leadership^9^,^28^,^29^. Finally, note that the high variation of directional accuracy in time (Fig. 2a) cannot be explained by a model in which carrying ants continuously reorient the load similar to the way a single ant follows a pheromone trail^30^.

Microscopically, we estimate the effect of an individual carrier by the change of load speed immediately following her attachment or detachment. In general, we find that ants at the leading edge of the object (Fig. 1c) tend to pull (evident by the recoil of the load on detachment, Fig. 2b, red), while those at the back assist the motion by lifting (see the non-specific direction change on detachment; Fig. 2b, green). Considering forces together with the ants' angular locations on the load and their carrying durations allows us to illustrate the typical time course of a carrier: on attachment, a new carrier tends to change the load's velocity towards her own pulling direction (Fig. 2b, blue). This influence lasts for several seconds (blue line, Fig. 2c) in agreement with the timescale of the initial improvement in load directional accuracy (Fig. 2a). The initial steering works to bring the carrier to the leading edge of the load (Fig. 2c, turquoise line), where she remains for about 20 s and continues pulling. At longer times that carrier's angular position becomes indistinguishable from that of any other ant (Fig. 2c, turquoise line) and the forces she applies decrease (Supplementary Note 8; Supplementary Fig. 5). In particular, the ant often ends up at the trailing edge of the load from where she cannot guide the motion. This provides strong evidence for the fact that all useful steering is provided by newly attached ants.

Multiplying the steering timescale of 5–20 s (Fig. 2a,c) by the mean attachment rate (Supplementary Note 9), we estimate the number of mean concurrent steering ants at 0.35–1.4. Figure 2d illustrates changes between these steering ants along one trajectory and demonstrates how a high turnover can bridge the scale gap between that of seconds, relevant to the effect of a single ant, and that of minutes, which characterizes the duration of the collective transport process (see Supplementary Note 10). The ants are able to compensate for the scarcity of information imposed by the small number of concurrent steering ants by maintaining high responsiveness to these ants, even as the number of carriers increases (Fig. 2e).

Although our results demonstrate that, over time, a large number of ants steer the collective motion, we do not claim that all ants are equal in their susceptibility to guide the motion or the degree of influence that they exert. Figure 2d shows that following attachment, some ants guide the motion for a longer duration than others. It is also possible that by detaching from the load and then reattaching to it an ant may extend her influence even further (examples for this are evident in Fig. 2d). It has been previously shown that the first *Formica schaufussi* ants to locate food are crucial for the transport process^29^. Differently, cooperative transport in *P. longicornis* relies on a larger number of transient steering ants and in Supplementary Note 11, we follow the recruitment process in this species and discuss its relevance to the subsequent transport (also see Supplementary Fig. 6).

Finally, the carrying ants' susceptibility to influence (Fig. 2a,e) seems to contradict the conformism^7^ that allows the ants to coordinate their forces and achieve high speeds (Fig. 1d). In the next section, we present a theoretical model that describes the cooperative transport and use it to reconcile this seeming contradiction.

### Theoretical model

**Figure 3:**
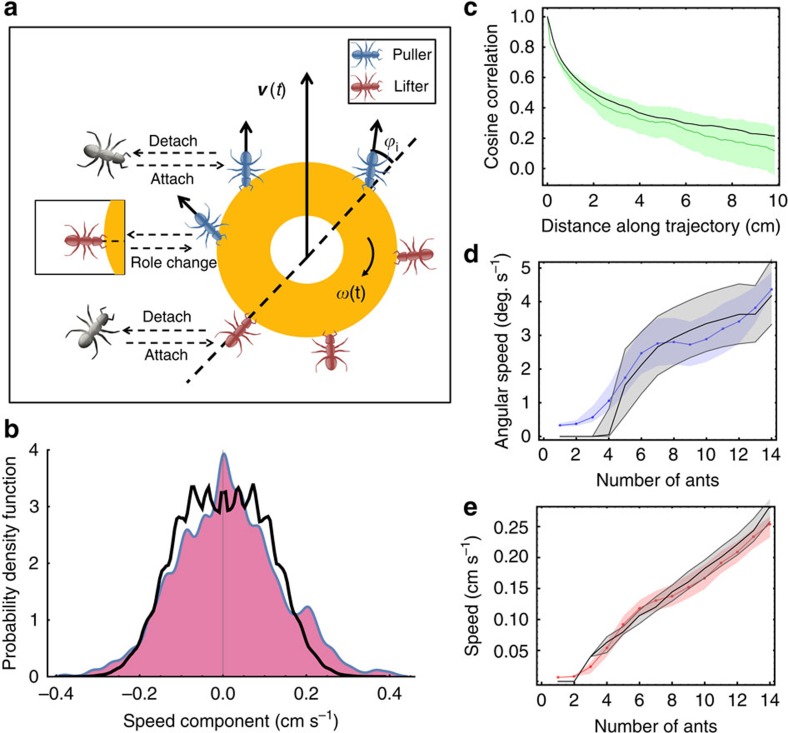
Microscopic model. (**a**) Model sketch including the possible transitions for non-informed individual ants. (**b**–**e**) The four model parameters were set by fitting experimental data of **b**. The distribution of the object's velocity (projected on an arbitrary direction) in periods of continuous motion (*N*=56,030 frames). (**c**) Correlation distance functions (*N*=17 trajectories). (**d**) Median speed (*N*=56,030 frames). (**e**) Median angular speed (*N*=56,030 frames). In each of these panels, the coloured lines represent the experimental data for ants transporting a load in the absence of informed ants. The solid black lines denote the results of our model. Error bars in **c** are the maxima and minima of correlation functions produced by partitioning the data into four parts. Error bars in **d** and **e** are the s.d. of a distribution of medians calculated for 1,000 samples bootstrapped from the data.

On the basis of the experimental properties outlined above, we constructed a theoretical model as specified in Fig. 3a, Supplementary Notes 12–14, Supplementary Fig. 7 and the Methods section. In short, the model is based on the minimal assumption that carriers interact uniquely through local forces transmitted to them by the load^31^. Informed ants are assumed to ignore these forces and attempt to pull the load in the correct nest-bound direction. Our experimental data suggests that the information held by ants deteriorates after they had been attached for a certain period (Fig. 2a,c). For simplicity, the model assumes that these ants become completely uninformed.

Non-informed ants either try to align their pull with the force vector on the load (but align against the local torque) or simply lift the object to decrease friction. An uninformed ant's decision of which role to perform is random and is biased by her alignment with the direction of the centre-of-mass force vector such that ants in the leading edge tend to pull (and ants in the rear tend to lift). In this way, the roles of the ants depend on their location around the load with respect to the direction of motion. An individuality parameter, *F*_ind_, determines the tendency of non-informed ants to randomly switch between pulling and lifting. A low individuality value indicates that an ant's decision is governed by her alignment with the group while a high value indicates random role switching, irrespective of the pulling force of the group. We denote the basal rate of this switching by *K*_c_. In addition, ants can attach or detach from the load. Finally, the object's speed and angular velocity were taken to be linearly correlated with the total force and torque as applied by all ants (Supplementary Note 12). The mathematical form of our model is motivated by models of collective transport on the subcellular^32^ and single-cell^33^ levels. While the population of ants may have a distribution of internal properties^34^, here we consider, for simplicity, a population of identical ants (for the behaviour of populations with non-uniform *F*_ind_; see Supplementary Note 15 and Supplementary Figs 8–9); in particular, the forces applied by informed and uninformed ants are taken to be equal.

We begin by comparing the model with the more elementary experimental condition from which informed ants are absent. This condition was achieved by carefully transferring the cargo along with the attached ants about 75 cm away from its initial location to a nearby clean board. Note that the relocation itself does not significantly affect the transport behaviour (Supplementary Movie 2). Under these circumstances, the ants-cargo system exhibits a persistent random walk (Fig. 3b,c; Supplementary Note 16; Supplementary Fig. 10). The model has four free parameters (see Methods): two (*F*_ind_, *K*_c_) are associated with the ants' decision-making process, while the other two are related to the mechanics of the specific ant-load system. We adjusted these parameters (Methods; Supplementary Notes 13,17 and 18; Supplementary Figs 7 and 11) to fit a large number of experimentally measured features (Fig. 3b–e). The good agreement demonstrates that the observed behaviours can be reconciled with a model that is based solely on mechanical information transfer; that is, the newly attached ant exerts its influence by causing a transient disturbance in the total force vector to which the rest of the ants react, all this without an active signalling mechanism^14^. Particularly, any form of influence must be implicit and is similar, in this sense, to the implicit influence that characterizes an effective leadership^13^. The sensitivity of the calculation to the chosen values of the free parameters is shown in Supplementary Note 19 and Supplementary Fig. 12.

### Balancing individuality and conformism

In the simulation, we define the response of the system to an informed, newly attached ant (Methods; Supplementary Notes 15 and 20) as the distance that the load travels towards the nest in the characteristic time between two consecutive attachments. Following Fig. 2a,c, we assume that an informed ant disorients, turning into an uniformed ant on a timescale of 10 s. This simple model assumes a discrete (but stochastic) process of forgetting. In Supplementary Note 15, we show that a more gradual forgetting process gives essentially the same behaviour, even when combined with a value of *F*_ind_ that is not constant over all ants (Supplementary Figs 9 and 13).

**Figure 4:**
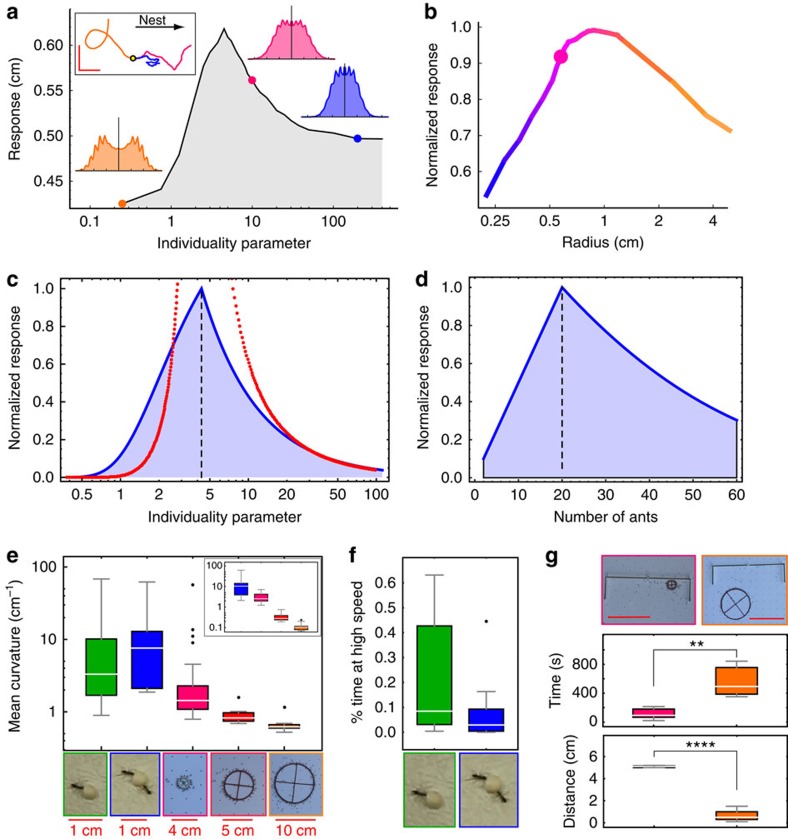
Group optimality. (**a**,**b**) Simulation data of the response of the object to a single attachment of a single-knowledgeable ant as a function of the individuality parameter *F*_ind_ (**a**) or of the object's radius (**b**). Insets depict velocity component distributions for small (orange) and large (blue) values of *F*_ind_ as well as its fitted value (pink). Upper left inset depicts trajectories (all starting at the yellow dot, colour coded as before) that take into account the continual arrival of informed ants (scale bars represent 10 cm). The pink dot in **b** marks the radius of the experimental load. (**c**,**d**) Exact solution of the Ising spin model. (**c**) Normalized (dimensionless) system response as a function of the individuality parameter *F*_ind_. The blue curve marks the short-term response to a newly attached ant and the red curve the mean-field susceptibility, which diverges at the critical point. (**d**) Normalized (dimensionless) short-term response to a newly attached ant as a function of the mean number of ants attached to the load (a proxy for load size). Dotted lines mark the critical transition points. (**e**–**g**) Experimental verification. (**e**) Mean absolute curvature of trajectories of objects of different size (total *N*=90). Synthetic and non-synthetic materials that were used for the small item exhibited similar curvatures (medians: synthetic: 8.84, non-synthetic: 6.53, unpaired two-sample *t*-test: *P*=0.6285, *N*=9) and were therefore pooled together. Thus, the effect presented in the figure is a size effect that cannot be attributed to load substance composition. Inset: mean curvature of simulated tracks of objects of different sizes (calculated on clean board conditions). (**f**) Time spent at *ν*>75% transport speed for one (green) versus 2–4 (blue) ants carrying a small load (total *N*=20). (**g**) Top: time to negotiate an obstacle (*t*-test: *P*<0.01) and (bottom) the maximal backwards displacement (*t*-test: *P*<0.0001) towards a successful crossing of a U-shaped block (which required 5-cm backtracking) for two load sizes (total *N*=11). Scale bar, 10 cm.

We fix three of the four free model parameters and check for possible optimality in terms of system response as a function of *F*_ind_. We find that both complete conformity (small values of *F*_ind_) and strong individualism (large values of *F*_ind_) reduce the effectiveness of a newly attached ant in steering the load (Fig. 4a). The fitted value of *F*_ind_ lies between these two extremes and this suggests that the ants operate in the transition region between strong and weak conformity, possibly to optimize their responsiveness to a limited influx of information (Fig. 4a, upper left inset). In addition, we find that the working regime of the ants is such that the velocity distribution of the load lies in the transition region between unimodal (tug of war or random walk) and bimodal (persistent motion) behaviours (insets of Fig. 4a).

Our model suggests a correspondence between load size and the ants' individuality (Supplementary Note 21). Namely, larger loads correspond to more conformist ants and vice versa. This behaviour naturally arises from the mean-field nature of this system (see below). We fixed *F*_ind_ to its fitted value and simulated ring-shaped objects of different radii (such that the mass per ant remains constant) to calculate the normalized response function to the attachment of a new ant. We find an optimal load size regime which is on the order of 1 cm (Fig. 4b). This scale is compatible with natural prey and nest entrance dimensions.

### Finite-size criticality

Figure 4a is reminiscent of the divergence of susceptibility near a second-order phase transition, and thereby suggests an analogy between *F*_ind_ and temperature, and between the response to a newly attached ant and susceptibility. To ascertain this analogy, we constructed a simple Ising model in which the group moves along one dimension (see Methods, Supplementary Notes 22–24 and Supplementary Fig. 14). This model is analytically tractable and its equilibrium state describes the centre-of-mass motion of the load. Note that although the model describes a one-dimensional (1D) motion the spin connectivity pattern has no spatial dimensionality (all spins interact with each other over a complete graph).

The spins in this Ising model denote the ants' roles: +1 for puller and −1 for lifter, while the external field is analogous to the force applied by an informed ant. Since all carrier ants are attached to the same cargo, each of them senses the total force exerted by all others and this makes the spin model inherently mean field. The mean-field solution (Supplementary Equation (41), which is an approximation for a finite-size system) reveals that, similar to the extended model described above, the response of the spin system to a transient pull by an informed ant (Equations (2) and (3)) peaks at *F*^c^_ind_ (*F*^c^_ind_=4.3 compared with the peak at *F*_ind_*=*4.25 in the extended model) for fixed *N* (Fig. 4c), and at *N*_c_ for fixed *F*_ind_ (Fig.4d). The critical value *F*^c^_ind_ indicates the transition of this Ising model between ordered (*F*_ind_*<F*^c^_ind_) and disordered (*F*_ind_*>F*^c^_ind_) spin states, where the order parameter is related to the mean speed (Supplementary Note 24; Supplementary Fig. 15). The susceptibility of the simplified 1D model diverges at the same critical point (Fig. 4c; Equation (4) in Methods). The good agreement between the critical points of the simple 1D model and the full two-dimensional model arise from the fact that rotations (which are absent in the 1D model) do not contribute to the ordering transition (Supplementary Note 24; Supplementary Fig. 16a), and in addition because reorientations of the ants make the two-dimensional system more 1D like (Supplementary Fig. 14).

Since the model is mean field, and unlike the typical behaviour of systems with short-range interactions, the critical point *F*^c^_ind_ increases linearly with *N* (see equation (1) in Methods). This unique property allows us to explore the phase transition by varying system size (Fig. 4b,d; Supplementary Fig. 16b) rather than *F*_ind_ (which is an inherent property of the ants and therefore difficult to manipulate experimentally). For example, large cargo sizes imply large *N* and therefore map to a large value of *F*^c^_ind_; since *F*_ind_ is fixed to some given value this implies that *F*_ind_*<F*^c^_ind_ and that, as a consequence, large loads are expected to be in the ordered (persistent motion) phase (Supplementary Fig. 17). Conversely, small systems have *F*^c^_ind_*<F*_ind_ and are in the disordered (random walk motion) phase.

### Experimental evidence for a mesoscopic phase transition

We used loads of varying sizes to experimentally test the models' predictions. First, our model predicts that ants can carry ring-shaped loads with arbitrarily large radii. Indeed, we could experimentally induce the ants to move a load of radius 8 cm, much larger than anything they naturally carry. We find that the curvature of the object's trajectory decreases with load size (Fig. 4e). The model shows the same dependence of the curvature with load size (Fig. 4e, inset); as the system becomes more ordered the curvature of the path decreases. Note that, in the case of small objects, a single ant involved in individual transport achieves a more direct trajectory than a small group of ants. This lack of coordination in small groups leads to non-optimal transport evident as a tug of war and a decrease in speed when compared with a single carrier (Fig. 4f). To demonstrate the suboptimality of a team of highly conformist ants, we used large (4 cm) radius objects that can occupy over 100 ants. In agreement with the model's predictions, these objects exhibit highly persistent motion (Fig. 4e; Supplementary Note 25; Supplementary Fig. 17). However, this large ant team was unable to traverse a U-shaped barrier placed in their path to imitate the ragged conditions encountered in the field (Fig. 4g; Methods; Supplementary Note 1). Note that smaller objects (1-cm radius) do pass the obstacle (Fig. 4g) and this is facilitated by the attachment of informed ants. This demonstrates, as predicted by the model, that ants lose their ability to exert positive influence in the case of large loads.

## Discussion

Cooperative load transport has been observed in 40 different ant genera^18^,^35^. To accomplish this impressive task, *P. longicornis* ants rely on some of the behavioural building blocks that were observed in these other species: attachment along the circumference of the load (encircling coordinated transport^1^), coordination of pulling forces such that the total force is increased^1^,^2^, individuals that take turns carrying the load^2^,^36^ and asymmetries between the influence of individual carriers^37^. In this work, we synthesize these building blocks into a coherent picture that sheds light on the behavioural algorithms employed by single ants^18^. Our work shows that *P. longicornis* ants optimize the efficiency of this collective behaviour in a way that works to minimize the duration of transport. Decreasing the time in which the food is exposed to external competition is believed^35^,^38^ to increase the ecological advantages of this group behaviour^18^.

The high degree of coordination required for collective motion demands efficient communication between carriers. The efficiency of communication depends, among other factors, on the connectivity network over which the messages are carried. There is an ongoing discussion regarding the communication network structures that are relevant for different animal groups (for example, bird flocks or fish shoals). Suggestions range from short-range interactions in which each animal responds to its nearest neighbours (either by metric^3^ or topological^39^ distance), all the way to long-range interactions that encompass large fractions of the group^40^,^41^. In the case described here, this ambiguity is, to some extent, lifted as the load imposes that all carrying ants respond to each other. The information that is being communicated on the network is also of interest. Contrary to some species that rely on the information and guidance of a single or few leaders^9^,^13^,^29^,^42^,^43^,^44^, we show that cooperative transport in *P. longicornis* ants is more distributed. Small amounts of information continuously enter the system as carriers detach from the load and make room for the attachment of informed individuals that correct the steering.

Aligning forces to effectively transport large items requires that carrying ants rely on public information. Using such second-hand information has potential disadvantages^45^. One problem is that social information is often less reliable than individually acquired information and that message quality can deteriorate even further as information passes between multiple individuals^6^,^46^. Since during cooperative transport all ants are strongly coupled, messages from the reliable source, a newly attached ant, are directly sensed by each carrying individual and this limits accumulation of errors. A second problem of social information usage is that it can lead to high conformity, loss of innovation and maladaptive collective behaviours^6^,^10^, these negative effects may increase with group size^7^. It has been shown that shoaling guppies may escape group pressure during particular instances in which they happen to break contact from the group^10^. Conversely, our model suggests that *P. longicornis* ants continuously balance conformism with individuality to optimize collective performance.

The intermediate level of conformity displayed by single ants places the group at a critical transition between two modes of collective movement. Criticality has been suggested as a unifying concept that underlies collective behaviour in biological groups^15^,^19^,^20^,^47^,^48^. It was proposed that groups of animals may evolve to function near a critical point, as this confers certain advantages such as increased responsiveness and the ability to process incoming information^47^,^48^. Unlike bird flocks^21^ and insect swarms^20^, where the groups have been suggested to be poised at the critical point independently of their size, we find that in collective transport by ants the critical point corresponds to a particular group size (Fig. 4). This unique dependence of the phase-transition point on the experimentally manipulable system size provides us with rare^19^, direct experimental evidence for critical behaviour in biological groups. Our results also demonstrate the effect that criticality has on the collective behaviour; we show how the universal physical phenomenon of divergence of susceptibility at the critical point is manifested as a peak in the group responsiveness. The fact that the immediate goal of the carrying group is readily evident, that is, getting the load to the nest, enables us to quantify the usefulness of the cooperative response. In this way, we quantitatively demonstrate how criticality facilitates the flow of useful information from transiently knowledgeable individuals to the carrying group^11^ (Fig. 2a).

When ants steer the load, it rotates so that they end up attached to its leading edge. This introduces a mechanical conflict between ants that happen to be attached at opposing ends of the load, even if they agree on the preferred direction of motion. This geometric constraint makes a ‘wisdom-of-the-crowds' type of opinion averaging strategy^49^,^50^,^51^,^52^ impractical in this case. Indeed, while ‘wisdom-of-the-crowds' improves with growing system size, here the opposite holds: obstacle avoidance capabilities deteriorate rather than improve when group sizes are large (Fig. 4g). Rather than averaging over all forces, cooperative transport in *P. longicornis* ants relies on the influence of one or a few newly attached individuals. These influential individuals cannot move the large load alone and the group serves to amplify their knowledge into a force that enables efficient cooperative transport of food items to the nest.

## Methods

### Experimental setup

Data were collected using five colonies of *P*. *longicornis* in Israel. Specifically, the number of experiments per each colony was 46, 25, 16, 6 and 5. Tests were carried out during the summer when these ants display collective transport behaviour^23^. Ninety per cent of the tests were carried out between 0900 hours and 1300 hours. All tests were carried out in conditions where temperature ranged between 26 and 30° C, with 79% of the tests performed at 26–27° C.

Experiments were conducted on a 100 × 70-cm board on which ants were allowed to cooperatively carry heavy loads. In each nest site, the testing board was positioned according to the availability of appropriate filming conditions (flat floor and a sufficiently large area with even illumination). The mean distance between the initial location of the food load and the nest entrance was 7.5 m with a minimum of 3 m (respectively, these are 2,500- and 1,000-fold the length of an ant). The board's dimensions were large, so as to allow the carrying team of ants to traverse long-distances uninterrupted.

Each filming day began with a recruitment phase, wherein a morsel of cat food was laid either nearby the board or on it, to attract ants. After sufficient recruitment, the cat food was switched with an object which was either a Cheerio or a 1.5-mm-thick ring-shaped piece of silicon. To make the objects attractive to the ants, they were first stored overnight in a closed bag of cat food (either Royal canin or Happy cat brands). Control tests in which Cheerios and silicon-made objects were placed in the testing area revealed that the ants have no interest whatsoever in these materials unless they were previously incubated with cat food. The carrying process was allowed to unfold without intervention, except initial placing on the board in some of the trials. On leaving the board, the team–load complex was carried back and released at various points on the board, including the original point. This procedure was repeated several times during each testing day. The entire carrying process was filmed at a resolution of 1,280 × 720@50 frames per second using a Canon EOS 550D camera mounted on a rolling stand. The board and load were marked with different colours to facilitate image analysis and tracking.

The data set used to fit model parameters was taken from another set of experiments (clean board) in which the team–load complex was picked up and then laid on a similarly marked 42 × 30-cm clean board, with no freely moving ants in its vicinity. The disturbance due to the repositioning was minimal (see Supplementary Video 1) and was excluded from the analyses. To ensure the board was not chemically marked, it was replaced after every experimental run.

Obstacle bypassing was tested using a U-shaped Perspex block (main text Fig. 4g). A small slit was cut in the middle of the U-block, allowing single ants, but not the load, to pass freely. This setup forced the carrying team to reverse their movement direction to pass. The time needed to bypass the block and the maximum distance travelled backwards were recorded. For further information see Supplementary Note 1.

### Image processing

A designated image processing programme was developed using MATLAB programming language. The programme's goals are consistent, high-quality tracking of the load's location and orientation, the ants' location, classification of ants as freely roaming or carrying, and tracking of the attachment points and body orientations of carrying ants. The programme's output includes full trajectories of the load and the ants in board coordinates, the load's orientation, ants' angular location on the load and ants' body orientation. This is achieved by recognition of the ants and the food item using their prominent features (such as circularity, colour and so on), and tracking the ants' identities. This linear assignment problem is solved using the Hungarian algorithm^53^, with a cost matrix based on distance between blobs. Automatic tracking fidelity is high, but not perfect. For calculations demanding completely precise identity data (for instance, data analysis related to attachments and detachments of ants from the load), manual correction of the data was performed. Camera rotation and translation are accounted for by tracking of the red spots marked on the board itself, so that camera motion may be backtracked and pixel location can be anchored to laboratory frame of reference. An algorithm classifying ants as carrying or freely moving with high fidelity has also been developed. This is achieved by identifying carrying ants to be those that are both in contact with the load and immobile in its frame of reference.

### Theoretical model

The model is described in detail in Supplementary Notes 12–15, 19 and 20 and illustrated in Supplementary Fig. 7. In short, the cargo is described as a ring with *N*_max_ empty sites. Each site is labelled by an angle *θ*_I_ (i∈[1,*N*_max_]) and can be occupied by a puller, a lifter or remain empty. Pullers exert force *f*=1 ant force while lifters reduce friction with a factor β·*f*. We sum all the forces that ants exert on the cargo and take the velocity to be linear in the total force (see Supplementary Note 12). Ants stochastically attach/detach from the cargo. Non-informed attached ants switch between pulling and lifting with rates 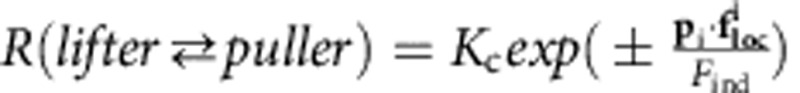, where the local signal force, 
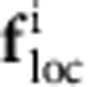
 (Supplementary Note 12), has contributions from both the velocity of the cargo's centre of mass motion and from the rotation around it, 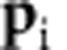 is the orientation of the ant on the cargo (see more details in Supplementary Note 12), *F*_ind_ is the individuality parameter, and *K*_c_ an internal decision-making rate. Informed ants differ from uninformed ants in that they always pull and do so in a direction that is as close as possible to that of the nest. Informed ants forget their knowledge and become uninformed with a rate *K*_for_ (for gradual forgetting see Supplementary Note 15). We use a Gillespie algorithm (see Supplementary Note 14) to simulate the stochastic dynamics of the cargo given the rates of attachments, detachments and decision making.

Figure 3 of the main text includes no informed ants as it corresponds to ‘clean board' conditions. Figure 4a,b include a single, informed ant. The inset of Fig. 4a includes a constant influx of informed ants.

### Ising model

This model simplifies the extended theoretical model into an analytically tractable description that nevertheless captures some important characteristics of the motion. We consider a system with a fixed number of attached ants, *N*, which is a reasonable approximation since attachment/detachment rates are smaller than the decision rate, *K*_c_, by an order of magnitude (Supplementary Note 13). As pullers tend to align towards a common direction, we further simplify the model to one where the motion is 1D and any dynamics are due only to role changing. These rates are given by 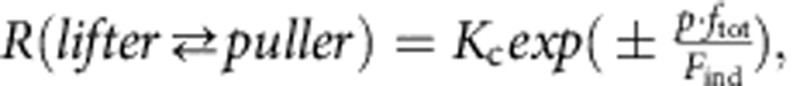 where *p* is ±1 for front/back. This yields an Ising-like model where the spin variable is +1 for a puller and −1 for a lifter with a temperature-like parameter *F*_ind_. As all ants interact with each other the system is mean field (Supplementary Note 22) and the critical value grows with system size


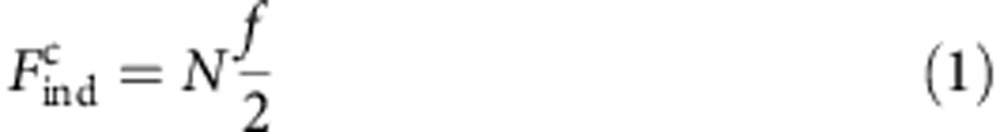


We also calculate the temporal derivative of the force when an ant becomes informed. The maximum of this response is at the critical point and shows a qualitative agreement with the results of the extended model (Fig. 4a,b). The transient response is given by,


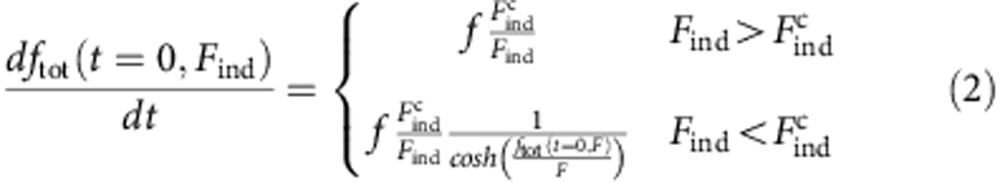


or as a function of *N*,


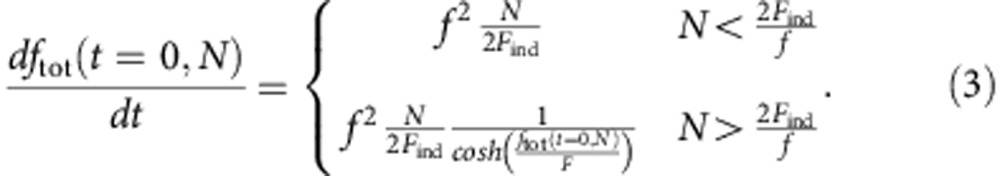


The mean-field thermodynamic susceptibility is,


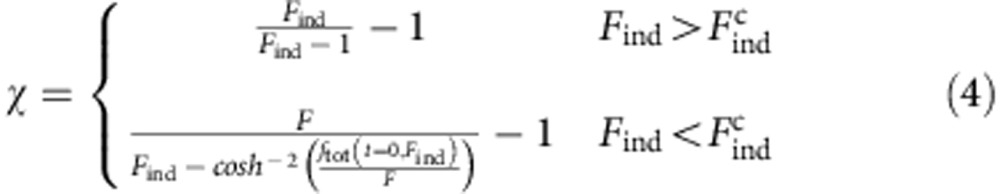


For a more complete description of the model and its solution refer to Supplementary Notes 22 and 23.

## Additional information

**How to cite this article:** Gelblum, A. *et al.* Ant groups optimally amplify the effect of transiently informed individuals. *Nat. Commun.* 6:7729 doi: 10.1038/ncomms8729 (2015).

## Supplementary Material

Supplementary Figures, Notes and References Supplementary Figures 1-17, Supplementary Notes 1-25 and Supplementary References

Supplementary Movie 1 A short clip of tracked cooperative transport. Tracking includes the location (blue dot) and orientation (unmarked) of the load as well as the trajectory of each individual ant within the frame (marked by the different colors). Ants are classified as either carrying (yellow number) or free (white number).

Supplementary Movie 2 Relocation test. This video shows an example of relocation test along the transport trajectory. During transport towards the nest, the carried load and attached ants were lifted by tweezers, gently brought back and placed at the initial point of the transport, located less than 1 meter back along the trail. Following the relocation only 1 out of the 15 carriers left the object while the rest of the group immediately continued the transport to the original direction towards the nest. This example suggests that the random-walklike behavior presented in the clean board experiments (Figure 3b,c and SI-S3.7) was not a response to the gentle relocation of the ants-object conglomerate but, rather, due to lack of relevant information.
