# Supplementary material for: Ant groups optimally amplify the effect of transiently informed individuals
Source: Nat Commun. 2015 Jul 28;6:7729. doi: 10.1038/ncomms8729 (PMC4525283; doi:10.1038/ncomms8729)
Supplement: Supplementary Figures, Notes and References — Supplementary Figures 1-17, Supplementary Notes 1-25 and Supplementary References [file ncomms8729-s1.pdf]

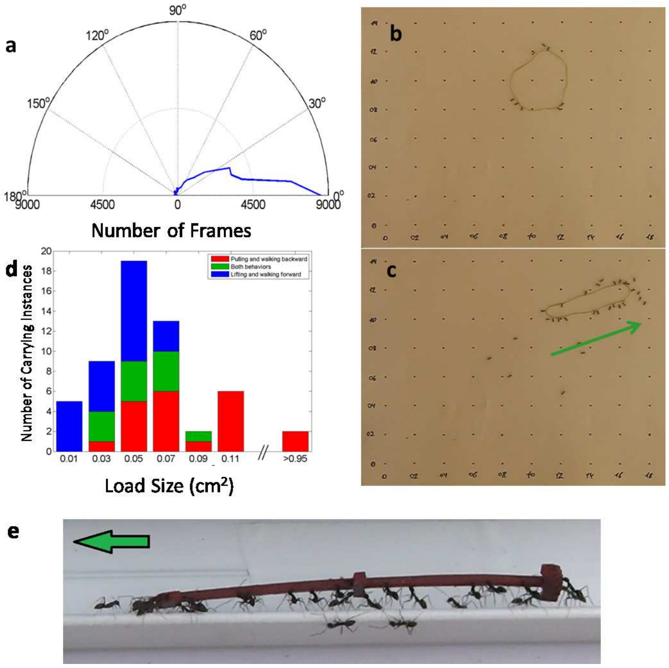

Supplementary Figure 1: Pushing vs lifting: **(a)** For every frame where all the ants were located within a section of 180°, the angular difference between load velocity direction and median ant angle on load was calculated. The figure shows a half-polar histogram of these differences. There is a clear bias towards 0, which implies the load is pulled and not pushed. **(b)-(c)** Experiments using elastic, locally deformable band as the carried object show distinct flattening of the band during collective transport, where the large axis of the resulting ellipse points in the direction of motion. The figure shows snapshots that were taken at **(b)** onset of motion and **(c)** during collective transport. Green arrow represents direction of motion. **(d)** Individually carrying ants adopt a new strategy when carrying large, heavy loads. Plotted is a stacked histogram of individual carrying instances for different load sizes. A clear transition from lifting and walking forwards (blue) to pulling and walking forward (red) can be observed. **(e)** A snapshot showing ants in the back of the load in a lifting posture. Green arrow represents direction of motion.

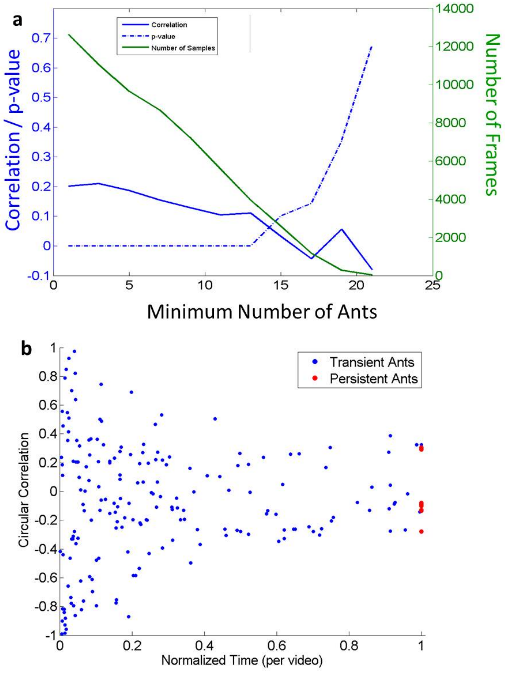

Supplementary Figure 2: Extreme cases of cooperative carrying: **(a)** No correlation between direction of motion and mean ant angular location on load: correlation coefficients between direction of motion and mean ant angular location were calculated for different number of minimum ants on the load (blue). Mean angular location is the vector sum of all angular locations in all frames within a given window ($\Delta t = 50\, frames = 1 sec$). For every non-overlapping window we thus get a mean ant angular location. The load velocity direction for each window is calculated using the formula $\mathbf{v} = \frac{\mathbf{x}\left(t+\frac{\Delta t}{2}\right)-\mathbf{x}\left(t-\frac{\Delta t}{2}\right)}{\Delta t}$, where $\Delta t = 50$, as mentioned above. The final value is simply the circular correlation between the mean angular locations and corresponding load velocities. As the minimum number of carrying ants rises, the distribution is necessarily more uniform, and so there is less correlation, which implies not all ants have the same importance in determining the direction of the load, given that ants do not push. Also shown are number of samples as a function of minimum number of carrying ants (green) and p-values of the corresponding correlation coefficients (dashed blue). **(b)** No despots exist in the carrying: each data point in the scatter plot corresponds to a correlation value between one carrying ants angular locations and the velocities of the load in the same frames, calculated as in (a). The correlation was calculated for the entire duration of carrying of each ant. This also includes cases where an ant attached and detached multiple times the correlation was calculated for all frames in which the ant was carrying. We plot the correlation as a function of the total time spent on the load (normalized to the duration of the experiments which ranged from 75 to 300 seconds). Every point corresponds to one ant. The red points correspond to ants which have carried the load for the entire duration of the experiment, whereas the blue points mark those ants which detached at some point from the load. The two groups are not statistically distinct (see main text), and thus, it is very unlikely ants which continually carry the load act as despots by enforcing their opinion on the group.

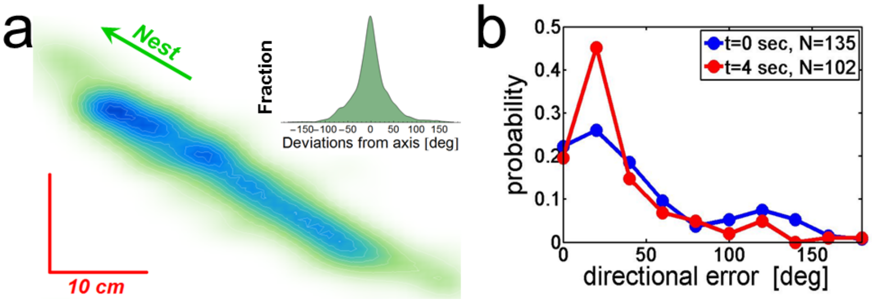

Supplementary Figure 3: Effect of newly attached ants, information and magnitude: **(a)** Heat map of overlaid trajectories of single ants that are heading to the nest (e.g. to recruit help). The inset demonstrates the tight distribution of heading directions around the nest-bound direction ($N = 34$ ants). **(b)** Velocity direction histograms: normalized histograms of load velocity directions at times $T = 0sec$ (blue) and $T = 4sec$ (red) relative to attachment of a new ant to the load. At time $T = 4$ the directional error of the load is usually smaller than prior to the attachment. Thus, the new ant injects information into the system, directing the entire group towards the nest.

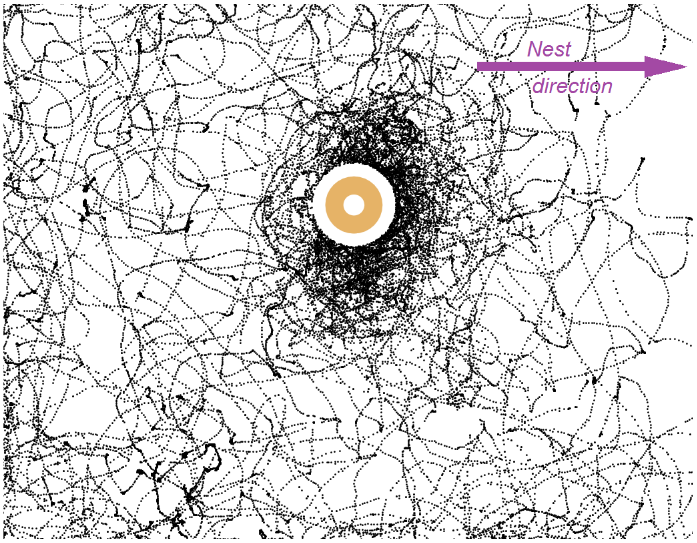

Supplementary Figure 4: Ants show no directional knowledge towards the nest after relocation of the load and carriers to a nearby clean area: Trajectories of detached ants during the first six minutes of a clean-board relocation test. The freely moving ants, while performing multiple loops originated at the load, explore all directions without any noticeable interest at the nest direction (purple arrow).

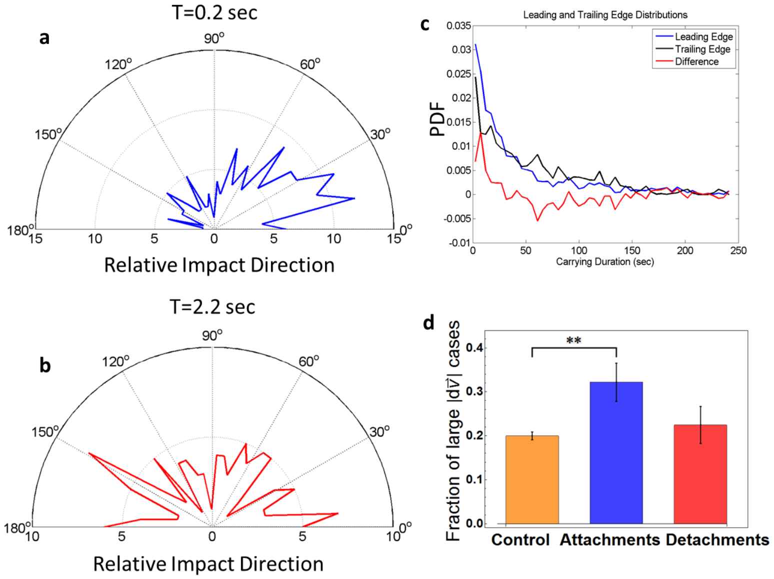

Supplementary Figure 5: Initial steering dynamics: two examples of half-polar histograms of the difference between attaching ant angular location and direction of change in load velocity (relative impact direction) at times **(a)** $T = 0.2sec$ (blue) and **(b)** $T = 2sec$ (red) relative to attachment time ($T = 0$). These two histograms illustrate the fact that as time passes, change in velocity becomes less and less correlated with the location of the attaching ant on the load. Thus, a characteristic timescale for initial steering can be computed. **(c)** Carrying duration distributions: normalized distributions of ant carrying durations over all frames, frames for front (blue, $-36°$ to $36°$) and back (black, $144°$ to $216°$) of load, relative to load velocity direction. The difference between these distributions (red) provides an upper limit bound for the timescale of influence in ants. **(d)** The forces applied by ants as they attach ($N = 115$) or right before they detach ($N = 98$) were estimated by the change in load speed as measured in the 2 seconds before and after these events. Plotted are the fractions of events with a velocity change larger than the 80th percentile of the control (no attachment or detachment, $N = 1987$). The distribution of velocity changes due to attachments is significantly different from that of control (Kolmogorov-Smirnov test on $|\mathbf{dv}| : p < 0.01$).

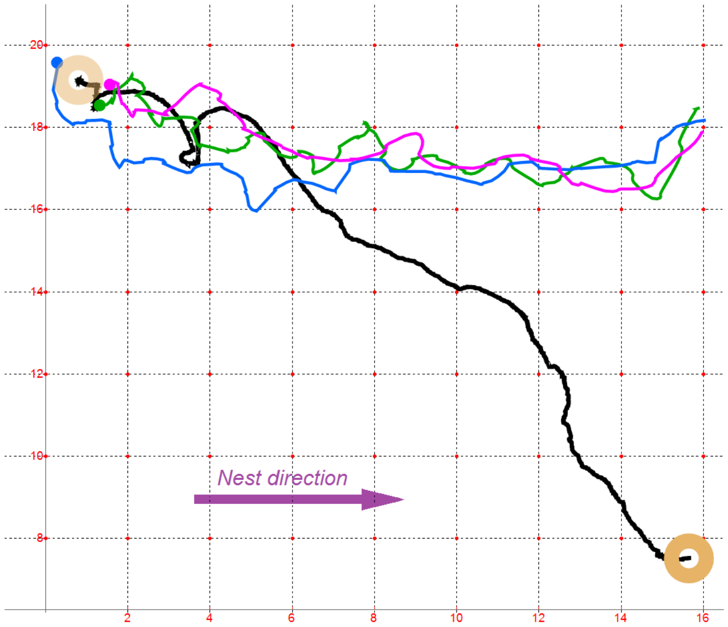

Supplementary Figure 6: Cooperative transport does not rely on the directional knowledge of ants during the preceding recruitment phase: After finding the food and prior to transport initiation, ants return directly to the nest for the purpose of recruitment. The trajectories of the first three recruiting ants (including the scout that initially found the nest) are depicted by the colored lines. The trajectory of the load, once it started moving, is marked in black. Trajectory of the load towards the nest significantly deviates from the paths of the initial recruitment trails. The load in this case never returned to any point on or near the initial recruitment trails on the 1m test board.

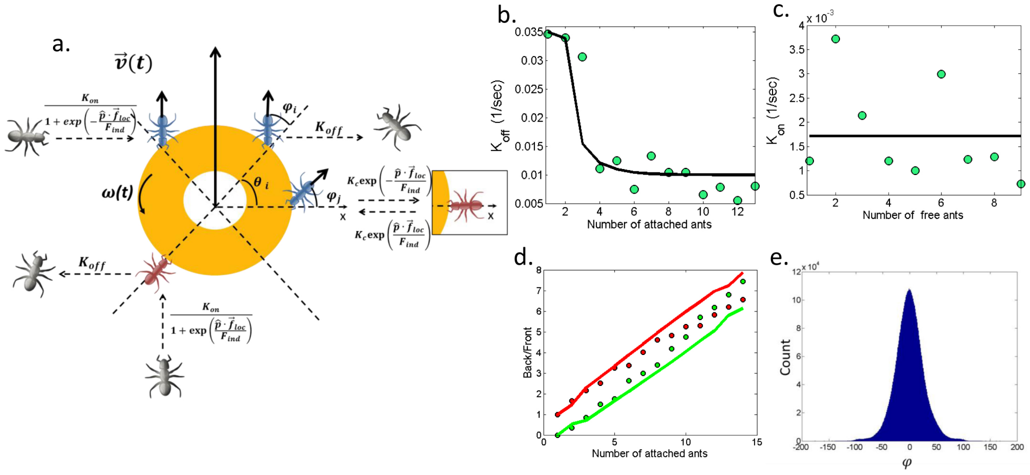

Supplementary Figure 7: Theoretical model details: **(a)** Illustration of the model. Grey ants are free ants, blue ants are pullers and red ants are lifters. Pullers try to orient with angle φ to the direction of the local force. Ants decide on their role and can change their decision while attached. **(b)** Circles - experimental detachment rates; black curve - model detachment rates. **(c)** Circles - experimental attachment rates; black curve - model attachment rates. **(d)** Occupation in the back (green) and the front (red) of the cargo vs the total number of attached ants. Circles correspond to experimental results and curves are for the model. **(e)** φ distribution - the orientation of the ants with respect to the radial direction on the cargo. The maximal φ is estimated as the standard deviation of this distribution which is 52°.

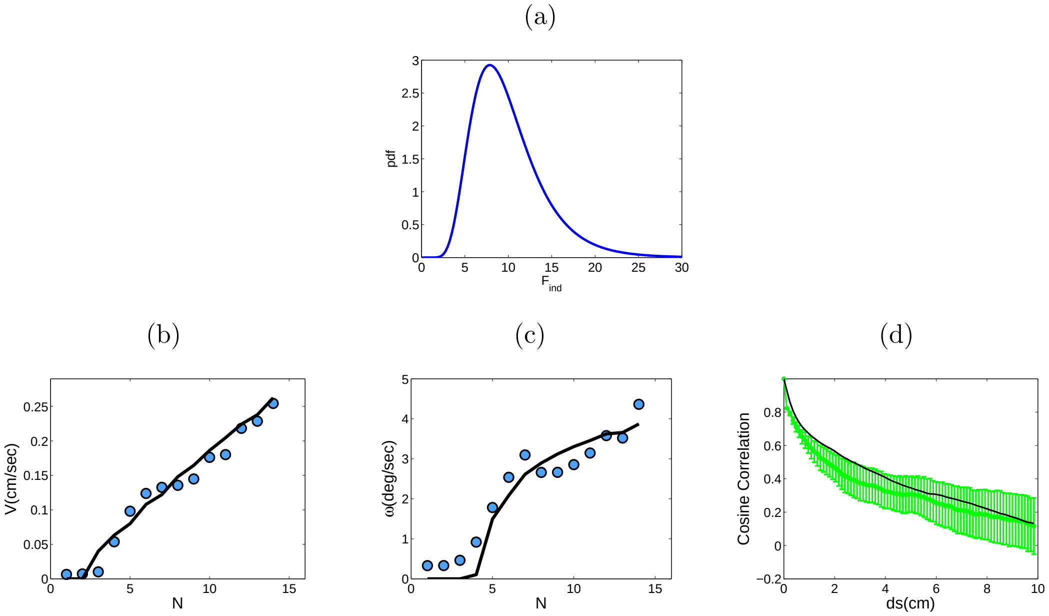

Supplementary Figure 8: Results for a model with a distribution of F_{ind} **(a)** Probability distribution function of the log-normal distribution with $\mu = 2.2226$, $\sigma = 0.4$ such that $\langle F_{\text{ind}} \rangle = 10$. **(b),(c)** - black curved represent the simulation results while circles represent the experimental results. In all the sub-figs $\langle F_{\text{ind}} \rangle = 10$. **(b)** Translational speed vs number of attached ants. **(c)** Angular speed vs number of attached ants. **(d)** Cosine correlation vs arc length. Green curve is the experimental results while the black curve is simulation results.

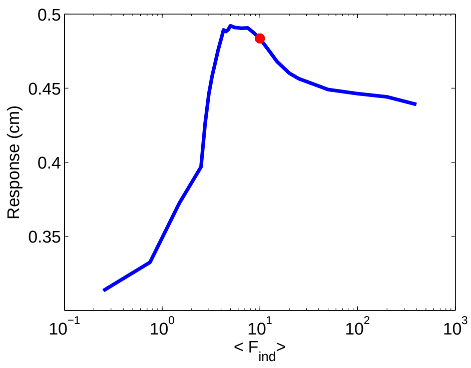

Supplementary Figure 9: Response for a system with a log normal distribution of F_{ind}s to an ant with gradual forgetting: Results shown in semilog scale. $\sigma = 0.4$ for all points in the figure. Red point represents the $\langle F_{\text{ind}} \rangle = 10$ as in Supplementary Fig. 8.

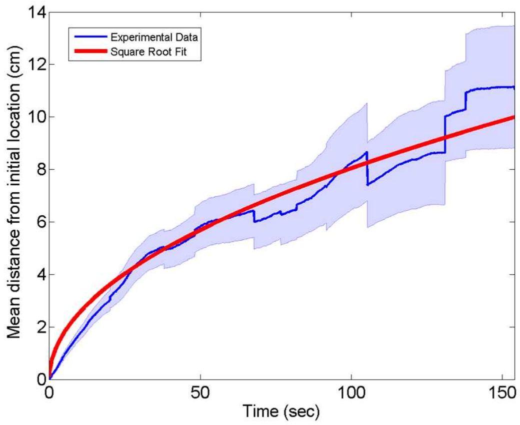

Supplementary Figure 10: Uninformed groups of ants engaged in cooperative transport exhibit random walk motion: The blue line represents mean distance as a function of time from commencement of motion, across 15 experiments. The blue shaded fill is the confidence interval, calculated using standard error. The thick red line is a square root fit of the data; using a model function $f = a\sqrt{t}$, we get $a = 0.805$, $R^2 = 0.942$.

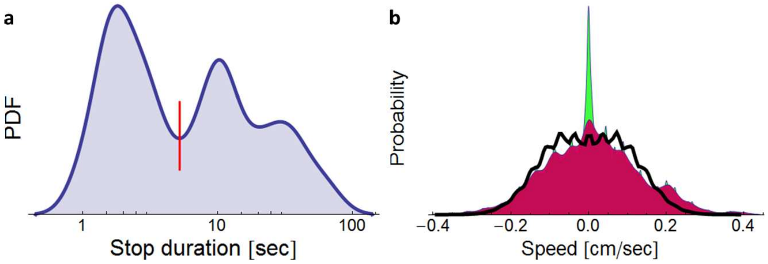

Supplementary Figure 11: Exclusion of no motion periods: **(a)** Distribution of stop duration in log scale. Red line ($T = 5.25sec$) marks threshold used for constraining speed data. **(b)** Constrained (red) overlaid on top of full (green) speed distribution projected on an arbitrary direction (opposite to nest direction, in this case). Black solid line is the model fit.

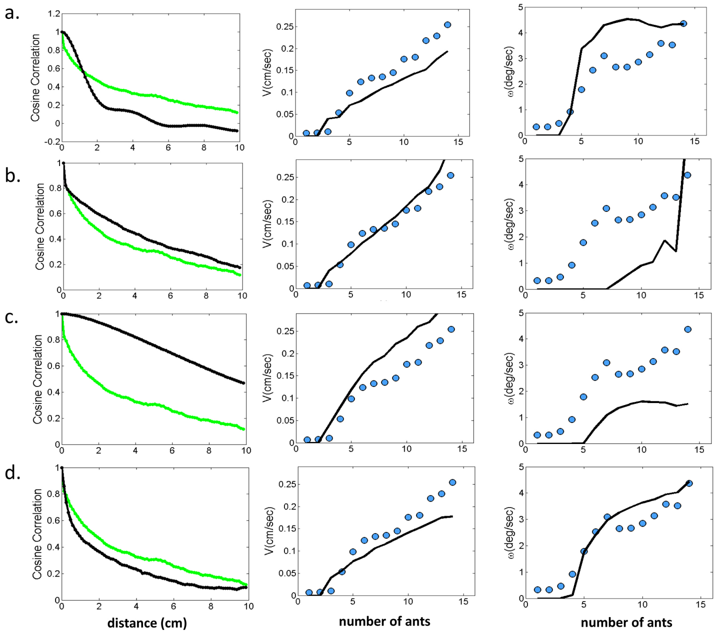

Supplementary Figure 12: Theoretical fits against experimental measurements for different values of the parameters K_{c} and F_{ind}: First column, cosine correlation, second and third columns, speed and angular speed as a function of the number of attached ants. Blue circles and green curves designate the experimental results and black curves are model results. **(a)** $K_{\mathrm{c}} = 0$, **(b)** $K_{\mathrm{c}} = 30 sec^{-1}$, **(c)** $F_{\mathrm{ind}} = 0.25$ ant force, **(d)** $F_{\mathrm{ind}} = 400$ ant force.

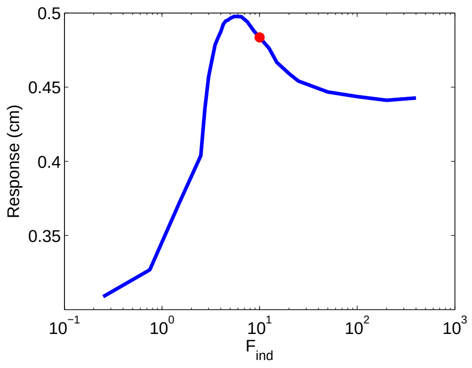

Supplementary Figure 13: Response to an ant with gradual forgetting in semilog scale: Red point represents the F_{ind} value of Fig. 3 in the main text.

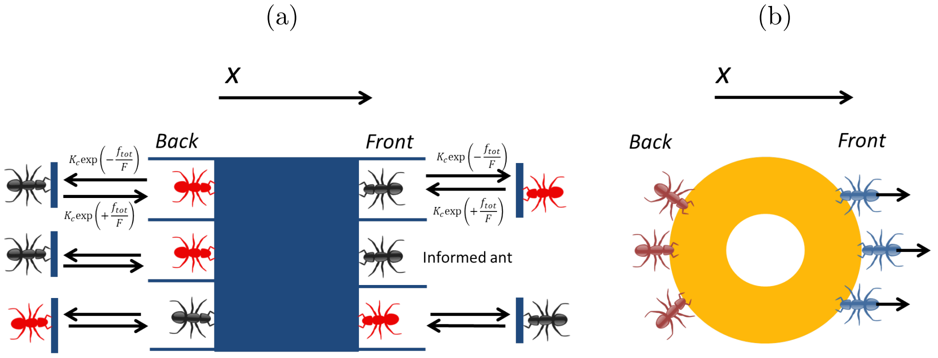

Supplementary Figure 14: Fully connected Ising model for motion in 1D: **(a)** Illustration of the Fully connected Ising model for motion in 1D with $N = 6$. The motion is only in the x direction in both directions. Black ants are pullers while red ants are lifters. Regular ants change their roles while the informed ant that knows the nest is in the positive direction remains a puller. **(b)** 2d model with reorientations can resemble a 1d model.

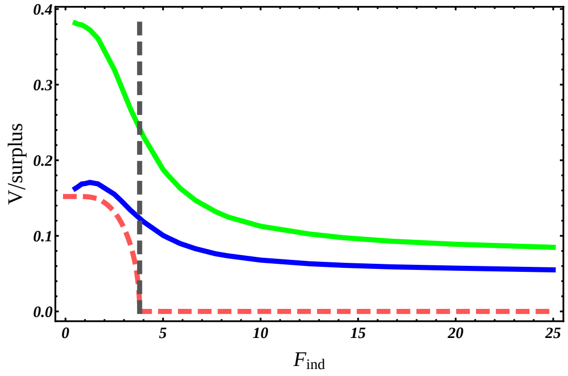

Supplementary Figure 15: Mesoscopic order-disorder curves for 1D system: Blue curve is the mean speed (equation (69)) while the green curve is the mean surplus (equation (70)) for a finite size system. Dashed vertical line is $F_{\rm ind} = 3.8$ while red dashed curve is the solution of equation (41).

(a)

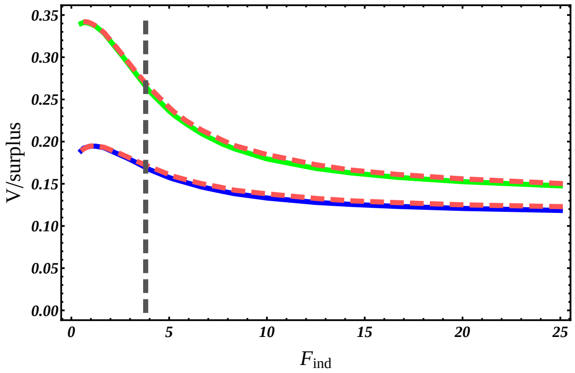

(b)

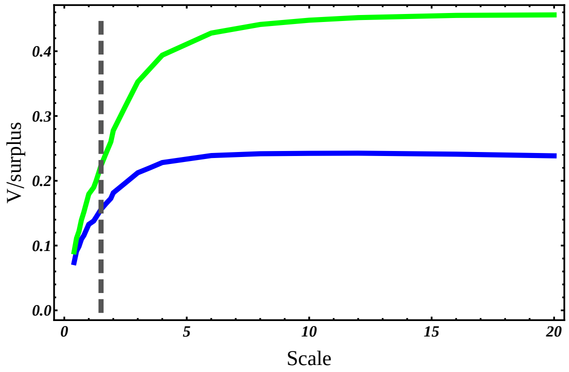

Supplementary Figure 16: Translational speed and surplus for the 2D system: **(a)** vs F_{ind} for a given size, Grey dashed lines denote $F_{\text{ind}}^{c} = 3.8$; red dashed lines correspond to a 2d system with no rotations; **(b)** vs size of the system for a given value of F_{ind}, Grey dashed lines denote the maximum in equation (68).

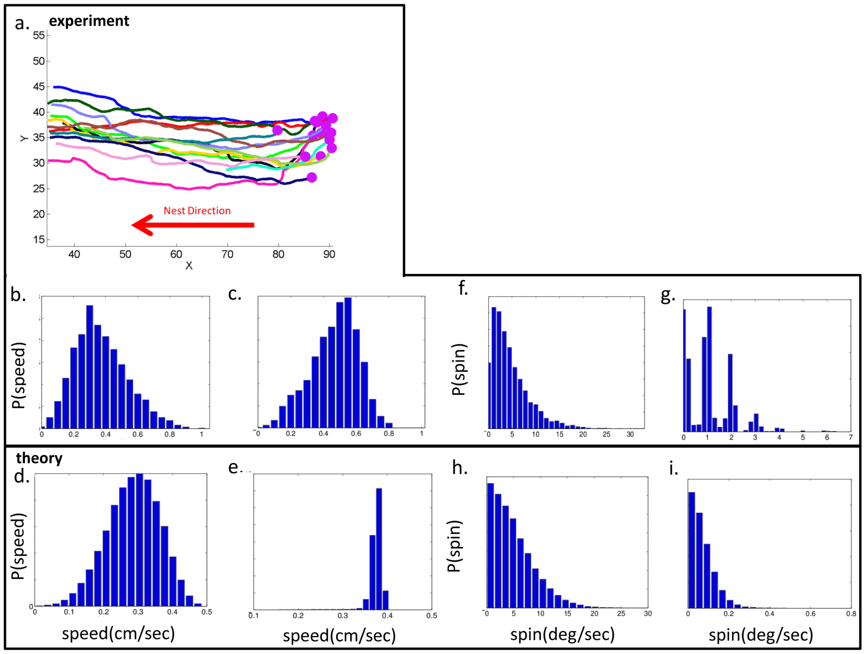

Supplementary Figure 17: Comparing large and medium load sizes: Large ($4cm$ radius) and medium ($1cm$ radius) objects exhibit different motion characteristics. **(a)** Examples of experimentally acquired large object trajectories ($N = 14$). The trajectories are smooth and have lower curvature in comparison to those of the regular size load. Purple filled circles are starting points of the trajectories. **(b)-(c)** experimental speed distributions; **(b)** medium size object,**(c)** large object. **(d)-(e)** model speed distributions; **(d)** medium size object, **(e)** large object. **(f)-(g)** experimental spin distributions; **(f)** medium size object, **(g)** large object. **(h)-(i)** model spin distributions; **(h)** medium size object, **(i)** large object.

Supplementary Note 1 Experimental setup

Data was collected using five colonies of *Paratrechina longicornis* located at Rehovot and Neve-Shalom (in the central area of Israel). Specifically, the number of experiments per each colony was 46, 25, 16, 6 and 5. Note that returning to the same colony does not guarantee that the same group of ants participated in the behavior: Each colony is composed of tens of thousands of individuals and one cannot control which of them join the transport on any specific day. One cannot even control this between different runs on a specific day. Tests were carried out during the summer due to the seasonal preference for high-protein diet during that time of year [1] when these ants display collective transport behavior. 90% of the tests were done between 9am and 1pm. All tests were done in conditions where temperature ranged between 26°C and 30°, with 79% of the tests performed at $26-27^o$C. In order to test the effect of temperature differences on the cooperative transport we compared the speed distributions and curvatures of load trajectories in the extreme temperature conditions tested. While load speed shows a 30% increase, the curvature of the load trajectory was not affected by ambient temperature (unpaired t-test, $p = 0.801$, $N_{\text{hot}} = 5, N_{\text{cold}} = 48$). Experiments used to measure load speed in Figs. 1d and 3e were all conducted in $26-27^o$C.

Experiments were conducted on a $100 \times 70cm$ board on which the ants were allowed to carry cooperatively. In each nest site, the testing board was positioned according to the availability of appropriate filming conditions (flat floor and a sufficiently large area with even illumination). The mean distance between the initial location of the food load and the nest entrance was 7.5 meters with a minimum of 3 meters (respectively, these are 2500 and 1000 fold the length of an ant). The board's dimensions were large, so as to allow the carrying team of ants to traverse long distances uninterrupted. To maximize usage of the board area, in all regular transport tests the load was placed $2cm - 10cm$ from the border of the far side of the board relative to the nest. This was done after preliminary tests that showed that starting point on the board did not change the clear preference of transport directionality towards the nest (see the trajectories examples presented in main text Fig. 1). In the clean-board relocation tests (see below), where the ants had no directional information on the board prior the resuming of transport, the load and attached ants were placed at the center of the board. The fact that the size of the board is much larger than the size of the load assures that most of the collected data comes from within the board and is not affected by its boundaries. Statistics for distance of the carrying ants from the borders of the board is as follows: For regular transport tests (large board): 96.7% $> 2cm$ (this scale is on the order of the size of load together with attached ants); 89% $> 5cm; 72\% > 10cm$. For clean-board test (smaller board): 99.6% $> 2cm$; 95% $> 5cm$.

Each filming day began with a recruitment phase, wherein a morsel of cat food was laid either nearby the board or on it, in order to attract ants. After sufficient recruitment, the cat food was switched with an object which was either a Cheerio^{TM} or a 1.5mm thick ring-shaped piece of silicon. To make the objects attractive to the ants, they were first stored overnight in a closed bag of cat food (either Royal canin^{TM} or Happy cat^{TM} brands). Control tests in which Cheerios and silicon made objects were placed in the testing area revealed that the ants had no interest whatsoever in these materials unless they were previously incubated with cat food. If the recruitment was done on the board, the carrying was allowed to continue without further intervention. However, if the recruitment was performed nearby, when the

group carrying commenced, the team-load complex was gently lifted using a delicate pair of tweezers and released on the board. Following placement, the transport dynamics were allowed to unfold without external intervention, besides the removal of foreign species which might interfere. In both cases, upon leaving the board the team-load complex was again lifted, carried back, and released at various points on the board, including the original point. This procedure was repeated several times during each filming session. The entire carrying process which took place on the board was filmed at a frame rate of 50 fps using a Canon EOS 550D camera mounted on a rolling stand. The experimental objects were colored on in advance, to facilitate image processing. The board was marked with a grid of small red spots of 1 mm in diameter with a spacing of 2 cm, and the board's edges were enumerated by cm, counting up from the bottom left corner. Together, these markings allowed tracking of the camera's movement and rotation. The load itself was marked with two spots of differing sizes opposite each other. These assisted in tracking the accurate location of the load's center and enabled acquisition of its orientational state.

The dataset used to fit model parameters was taken from another set of experiments. In these, after the initial recruitment phase, which was done about 75cm away from the filming area, the group of carrying ants were picked up and gently laid on a 42×30cm clean board, with no freely moving ants in the area prior to the repositioning. The disturbance due to the repositioning was minimal (see Supplementary Video 1) and was excluded from the analyses. The carrying dynamics were then allowed to unfold. The board was replaced for each experiment run. These measures were taken to ensure that the board was not chemically marked by the ants in any way before each trial was initiated. Markings on carried item and board were the same as in the experiments described above. For testing how load size affects the ability of the ants to bypass an obstacle that requires backwards carrying (main text Fig. 4g) we used a U-shaped Perspex block (base length: 20 cm, "legs" length of the U-block were 5 cm each). Block height was 2 cm and 0.3 cm thick. In order to make the task more difficult a small slit (1 cm height and 0.5 cm wide) was cut at the middle of the base of the U-block. In this way single ants were able to pass freely but the carried loads were not ($R = 1$ cm and 4 cm). The U-block was positioned such that it would block the carried load by placing the obstacle perpendicular to the estimated trajectory of the carried load and with the "legs" of the block pointing backwards towards the approaching load. This set-up ensured that in order to bypass the block the ants would have to reverse their movement direction at least for 5 cm. To quantify the flexibility of the system for such direction reversal 2 variables were measured: i) The time the ants needed to bypass the block measured from the first touch of the carrying group at the base of the block until the successful bypassing of the block (or abortion of test after long duration times due to technical issues, for the large loads), and ii) The maximum distance the load traveled backwards after encountering the obstacle, measured from the base of the block.

Supplementary Note 2 Error estimation

Throughout the analysis, error estimations for median values were calculated using bootstrapping techniques. First, the original data was bootstrapped, creating 1000 new samples from the original sample. From these samples, a distribution of medians was calculated. The

16th and 84th percentiles of this distribution were taken as the lower and upper error size, respectively.

To estimate errors for calculations of mean values, standard error of the mean was used symmetrically, as given by $Error = \sigma/\sqrt{N}$.

In main text Fig. 3c, errors were calculated in the following manner: first, the data was segmented into 4 parts. Then, the correlation function of each part was calculated. By comparing these four functions $\{f_i(x)\}$, minimum and maximum values for each arc length value were obtained. The symmetric errors used in the graph are simply $(\max(\{f_i(x)\})$-$\min(\{f_i(x)\}))/2$, for every value of arc length x.

Supplementary Note 3 Pushing vs. lifting

Pushing behavior in collective load carrying of *P. longicornis* groups is rare to nonexistent. Four pieces of evidence support this claim. First, for cases where all carrying ants happen to be located within a section of 180° there is high correlation between median ant angle and direction of motion of load (Supplementary Fig. 1a) which indicates pulling. Note that we have found no instances in which this collective pulling state commenced with a pushing stage which, via some mechanical instability and the object's rotation, transitioned into pulling. Rather, when all ants are located on the same hemisphere of the object pulling commences immediately. This implies ants tend to carry heavy loads by pulling, and that pushing is much less probable. Second, more direct, albeit qualitative, evidence comes from experiments performed with a thin elastic band. In these experiments one can observe local deformations in the band due to forces applied by the ants. As the carrying process progresses, the initially round elastic band becomes eccentric with the large axis parallel to the direction of movement, suggesting the ants in the front pull while the ants in the back do not push (Supplementary Figs. 1b,c). Third, observations of individual ants carrying (relatively) heavy loads show a transition in carrying strategy from lifting and walking forward to pulling and walking backwards (Supplementary Fig. 1d). Individually carrying ants do not exhibit pushing behavior at all. Finally, while detachment of ants from the leading edge of the load lead to change in velocity direction opposite to their angular location on the load, change in velocity direction after detachments of ants from the trailing edge is spread uniformly (main text Fig. 2c). This implies ants in the front pull, while ants in the back do not push. Taken together, we conclude that pushing as a strategy for collective load transport is not within the behavioral repertoire of these ants. Thus, the only remaining option, given the effect of ants in the trailing half of the load on its velocity (main text Fig. 1d) is lifting, possibly, to reduce friction.

Supplementary Note 4 Individual ant force

The order of magnitude of forces exerted by individual ants was estimated using two procedures:

1. Taking instances where carrying ants lost their grip on the load and detached forcefully, their pulling force can be estimated using $Fx = mv^2/2$, where F is the force exerted by the ant, x is the length of the ant's leg - an upper bound for the distance over which

she applied her force, m is the ant's mass and v is the velocity magnitude just after detachment.

2. The heaviest object a single ant can move was chosen. The experimental board was tilted vertically until the object began sliding down. Using this critical angle θ, an estimate for the force F needed to move the load from its static position can be obtained using the equation $F = mg\sin\theta$, where m is the ant's mass and g is earth's gravity.

Supplementary Note 5 Extreme cases of collective carrying strategies

Collective decision making arises through integration of individual opinions. The manner of this integration varies between different phenomena, ranging from one extreme case where all individuals have equal weight, to the case in which one individual dictates the future course of group action.

As seen in the previous note, in our system ants pull backwards to steer the load. This means a convergence of directional opinions predicts a correlation between mean ant angular location on the load and its direction of motion. When the carrying group is small, most ants are located on the leading edge of the load, and thus there is some correlation; however, as the number of carrying ants grows, their angular distribution around the load becomes more and more uniform, which inevitably leads to weaker and weaker correlations (Supplementary Fig. 2a). This suggests the observed collective transport is not a result of a "wisdom-of-the-crowds" type of opinion averaging.

On the other edge of the spectrum lies the case in which specific ants dictate the global motion, resulting in very high correlations between the angular location of these ants on the load and its direction of motion. Such ants must be attached to the load for the entire duration of carrying. However, we find that correlation between ant angular location and load motion direction is not a function of time spent attached to load (Supplementary Fig. 2b). Importantly, ants which are attached for the entire duration of the carrying have low correlations, and are not distinct from other ants. This was verified by performing an unpaired two-sample t-test on the correlation coefficients of the two groups, resulting in $p = 0.669$. The data was collected from 5 experiments. Number of ants considered to be potential persistently influential individuals (attached for the entire duration of the carrying) was $N_{\text{persistent}} = 14$ ants (4, 3, 2, 4 and 1 ants in the different experiments); Number of ants attached for only a part of the carrying was $N_{\text{non-persistent}} = 186$ ants (30, 22, 32, 51 and 51 ants in the different experiments).

Supplementary Note 6 Quantification of directional information

Figure. 2a of the main text quantifies the amount of information injected into the system by newly attached ants. These ants were recently unattached and therefore possess information regarding the correct direction to the nest (Supplementary Fig. 3a). This is calculated from the entropy of the histograms of velocity directions as a function of time from attachment of a new ant to the load. For each ant attachment, directions of velocity (relative to general direction of load motion) at times T relative to attachment time ($T = 0sec$) were calculated. For every time delay T, a histogram of velocity directions was created, and its entropy was

calculated. Examples of such histograms, corresponding to times $T = 0sec$ and $T = 4sec$, are plotted in Supplementary Fig. 3b. From these histograms information was calculated using $I(X,Y) = H(X) - H(X|Y)$, where X is the random variable that holds the instantaneous angle between the load and the nest, Y is the random variable of the load's direction of movement, $H(X)$ is the entropy of the uniform distribution, $H(X|Y)$ are the conditional entropies of the velocity direction histograms, such as those depicted in Supplementary Fig. 3b, and $I(X,Y)$ the amount of directional information in the system. Clearly, at $T = 4$ sec the load is better directed towards the nest than at $T = 0sec$, implying injection of information by the recently attached ant. Errors in Fig. 2a of the main text were generated by creating 10000 histograms for each time delay T, where bin i takes a value $\tilde{q}_i = q_i + \beta_i \sqrt{q_i}$, where q_i is the value of the bin in the corresponding original histogram and $\beta_i \in (-1, 1)$ is a random noise term. From these histograms, a distribution of entropies was generated for each time delay T. The standard deviations of these distributions were taken as the errors of the entropy measurements, and thus of the information.

Supplementary Note 7 *P. longicornis* ants do not rely on visual cues to navigate home

In order to examine the possibility that these ants rely on visual cues we compared relocation tests where the carried object and attached ants were lifted by tweezers and gently placed in two visually similar conditions: i) back along the trajectory of transport and ii) to a near-by clean board. In both tests the new position of the load was less than 1 meter away from the original location allowing clear view of the area and practically keeping the same lines of sight as in the original location. The results show that whereas in the first case the ants immediately resumed the transport of the load towards the original direction of the nest (see example in Supplementary Video 2), in the clean-board test the carried load meanders, and exhibits a random-walk-like movement pattern (Fig. 3b,c, Supplementary Note 16 and Supplementary Fig. 10). In addition, the freely moving ants (i.e. non-carrying ants) in the clean-board test, while performing multiple loops originating at the load, explored all directions without any noticeable interest at the nest direction (Supplementary Fig. 4). This behavior has little resemblance to the highly oriented movement of recruiting ants that tend to return directly towards the nest (Supplementary Fig. 6). All these findings support our view that these ants do not rely on visual cues not only during transport when the ants are hooked-up to the load but also when the ants are free to travel around.

Supplementary Note 8 Directional influence timescales

The dynamics of influence of specific individuals are plotted in main text Fig. 2c. The blue line represents an initial stage of steering by newly attached ants. For each newly attached ant, we measured the angular difference between the location of the ant and the direction of change in load velocity at different time delays T, from the moment of attachment of the new ant $T = 0$. For every time delay T, a half-polar histogram of these angles was calculated (two example histograms are shown in Supplementary Fig. 5a,b). The blue line is the fraction of occurrences between 0° and 45° (above chance), as a function of time that

has passed since the moment of attachment at $T = 0$. The timescale for steering by newly attached ants is thus demonstrated through the dynamics of the relation between direction of change in load velocity and angular location of recently attached ants. The timescale for the initial steering phase represents a lower bound on the entire duration of influence; after the ant has steered the load in the preferred direction, it continues exerting force and pulling the load from its position on the leading edge, but steering is no longer needed.

An upper bound for the leading effect timescale, where the newly attached ant has reached the leading edge of the load and continues pulling in the direction of the nest, we've estimated from the angular distribution of ant carrying durations over the load. As weve seen, recently attached ants (”young” ants) steer the load and determine its direction by pulling. These influential ants do so from the leading edge of the load. On the other hand, ants which have been attached for longer periods (”old” ants) do not control their location on the load, and so they are dispersed equally over the entire load. These ants no longer contribute significantly to the load's motion direction. Using this information we can estimate the duration ants spend on the leading edge after their attachment and deduce an upper bound for the influence timescale. The distribution of ant carrying durations in the leading edge of the load includes both young ants (which we identify as influential) and old ants, whereas the distribution on the trailing edge would consist mostly of old ants. The difference between these distributions corresponds, therefore, to the presence of young influential ants in the leading edge of the load. Specifically, the distributions of ant carrying durations as a function of angular location on load (relative to velocity direction) were calculated. From these, the carrying duration distributions in the front ($-36°$ to $36°$) and back ($144°$ to $216°$) were extracted (See Supplementary Fig. 5c). The turquoise line of Fig. 2c in the main text depicts the difference between the normalized versions of these distributions. As noted above, we interpret this difference as the result of leading ants' presence in the front edge and thus we get an upper bound estimate for the average time an ant spends actively directing the motion of the load. The decrease in pulling force as the time since attachment increases is further demonstrated by the reduced effect on load speed as caused by detaching ants when compared to attaching ants (Supplementary Fig. 5d).

We note here that, in principle, the influence of an ant may decrease with time not because she loses knowledge but rather her capacity to communicate it. It is difficult to experimentally distinguish between these two possibilities since this would require looking into the brain of the ant. Fortunately, on the level of the group, these two alternative explanations are completely equivalent and distinguishing between them is of secondary importance.

Supplementary Note 9 Estimation of number of concurrently steering ants

To estimate the average number of concurrent ants influencing the direction of motion, we used the following formula $N_{\text{steer}} = A \cdot F_{\text{steer}} \cdot \tau$, where A is the mean rate of attachment for the regular (non “clean sheet”) experiments, F_{steer} is the fraction of steering ants from the total number of newly attached ants, which is taken from Supplementary Fig. 5a, and τ is the timescale of persistent steering calculated from Main text Fig. 2c. Plugging in the appropriate numbers we get $N_{\text{steer}} = 0.2 \cdot 0.34 \cdot [5 - 20] = 0.34 - 1.36$.

Supplementary Note 10   Steering dynamics example

Figure 2d in the main text illustrates changes in influence of steering ants along a single trajectory. Since not all attaching ants are equally influential, since influence duration varies widely, and since some different strategies for leading exist (rotations vs. radial pulling, pulling hard in the beginning vs. pulling persistently) this figure was produced by applying several selection criteria on all carrying ants. The criteria are designed to include ants which incur relatively large changes in the motion of the load upon attachment or ants which are consistently located on the leading edge. It is important to stress that even tough the following criteria follow the rules deduced from the observed phenomena they do have an arbitrary component. Furthermore, some potential influential individuals were excluded because of technical limitations. Thus, the resulting list of influential individuals is neither complete nor entirely accurate, yet it gives a rough estimate which is good enough for illustration purposes. The criteria used were:

1. Ants which are located in the front for a long duration most probably have an effect on the loads direction: Ants which are located between - 60° and 60° (relative to load velocity direction) for the first 8 seconds of their carrying (or for the entire duration of their carrying, if they carried for less than 8 seconds).

2. (a) The more ants spend in the front, the more they are likely to be influential: If the entire carrying duration T is such that $3sec < T < 12sec$, then time spent located between 60° and 60° must be greater than $T/2$; If $T > 12sec$, then time spent located between - 60° and 60° must be greater than 6 seconds; T must be greater than 3 to pass this criterion.
   and (

   (b) In some cases the influential ant mainly rotates the load upon attachment:
   $d\omega > 6°/sec$
   or

   (c) In other cases influential ants have a significant effect on the velocity of the load upon attachment:
   $\frac{dv}{v} > 0.5$

   (d) and
   Excluding cases where initial load velocity was very low, thus rendering the dv/v criterion meaningless: $dv > 0.05(cm/s)$ or $dv > 0.1(cm/s)$ (in case the ant carried for less than 20 seconds). If the ant carried for a short while, than to be considered influential she needs to have a stronger initial effect.
)

The criteria used for deciding when an ant stops being influential were:

If an influential ant was located on the large section between 45° and 315° for more than a second in total, or if it detached prior to meeting this criterion, it stops being influential.

Supplementary Note 11 Influential ants and the recruitment stage

In their work on collective transport in *Formica schaufussi*, [2] have found that the scout that first founds the food and recruits to it is absolutely required for collective transport to occur. Recruited ants, even if they reach the food, do not carry or recruit to it. In that paper the scout that first found the food is termed a transient leader, where the word "transient" refers to the duration of a single transport event. In other words, when a different scout locates a new piece of food then she becomes the new (transient) leader.

Note that this use of the word *transient* is different from what we refer to in this work in which transient refers to the very short time period (on the order of 10 seconds) following an ant's attachment to the load in which she makes a positive effect on its directionality. However, it may still be the case that by repeated detachments and re-attachments a single ant may be able to influence the load directionality for extended periods of time (see examples in main text Fig. 2d). In particular, we check whether, *P. longicornis* rely on the first ant that found the food to lead the object (similar to *Formica schaufussi*). Figure 2d in the main text shows about 50 cm of trajectory in which the number of unique leaders was on the order of ten. This is more than the single scout that first found the item so it cannot be the case in which only she can steer the load. Another piece of evidence supporting the fact that the first ants that have reached the food are not those that determine the trajectory to the nest is provided in Supplementary Fig. 6. This figure shows the routes that the first three recruiting ants have taken on their way from the load to the nest. Overlaid on this is the route that the object itself took once it started moving. It is easy to see that these routes quickly diverge such that the trajectory that the load takes to the nest relies on different information than what was conveyed by the initial recruiters.

Finally, we counted the number of ants that reached the area of the food before it actually started moving. This number is quite large and stands between 54 ± 13 (mean $\pm$ SEM of maximal number of ants near the food at a single point in time, $N = 13$) and 143 ± 29 (mean $\pm$ SEM of the sum of all ants near the object such that an ant that leaves the frame and returns will be counted twice, $N = 13$). It is therefore absolutely possible that all subsequent influential ants come from this group.

Supplementary Note 12 Description of the theoretical model

Here we describe the rules of the theoretical model. Consider a circle (the cargo) and divide it to N_{max} equally spaced sites labeled by the angle θ_i, $i \in [1, N_{\text{max}}]$. Each site can be either occupied with a puller, a lifter, or be empty. The cargo is surrounded by N_{av} ants (which is the reservoir from each ants can attach to the cargo, see equation (15)); the number of empty sites is denoted by N_{empty}. When a puller ant is attached to the cargo she contributes to the cargo's velocity by applying a force, and gets aligned as much as possible with the direction of the local force at its point of attachment. The angular orientation of a carrier with respect to the local outwards normal at position θ_i is denoted as φ_i. Pullers pull the cargo while lifters lift it to reduce the friction by a factor $\beta \cdot f_0$ (where f_0 is the magnitude of the force an ant exerts). The model is illustrated in Supplementary Fig. 7a.

If a puller ant overcomes the static friction then the velocity of the center of mass $\mathbf{V_{cm}}$,

and the angular speed ω, are given by:

$$\mathbf{V_{cm}} = \frac{\mathbf{f_0} - \mathbf{f_{kin}}}{\gamma}$$
$$\omega = \frac{\tau_0 - \tau_{\text{kin}}}{\gamma_{\text{rot}}} \tag{1}$$

where $\mathbf{f_0}$ (τ_0) is the force (torque) exerted by a single puller, $\mathbf{f_{kin}}$ is the kinetic friction (with τ_{kin} as the kinetic friction torque), $\gamma = M/\delta t$ is the cargo response coefficient for a force applied over time δt and γ_{rot} is similarly the response for an applied torque. The rationale for these linear relations is as follows: The motion of the cargo is composed of many pulling events by the ants. We assume that the ants effectively pull synchronously over a short time period δt. From Newton $2nd$ law we write:

$$M\frac{\delta \mathbf{V_{cm}}}{\delta t} = \mathbf{f}_{\text{tot}} \tag{2}$$

or,

$$\delta \mathbf{V_{cm}} = \frac{1}{M/\delta t}\mathbf{f_{tot}} \tag{3}$$

where $\delta \mathbf{V_{cm}}$ is the velocity of center of mass (or $\mathbf{V_{cm}}$) as the cargo is at rest when the ants begin to pull it. Since the ants perform the pulling at a high rate we treat the velocity as continuous and neglect the discrete nature of the pulling events. In this picture we neglect the inertia of the object as the friction slows down the cargo velocity after the ants stop to exert forces. We write a similar equation for the angular speed in the following note.

The velocity components of the cargo are sums over the contribution of pullers and the friction:

$$V_{\text{cm,x}} = \frac{f_0 \sum_{i=1}^{N_{\text{max}}} n_{\text{p}}(\theta_i, \varphi_i)\cos(\theta_i + \varphi_i) - f_{\text{kin,x}}}{\gamma}$$
$$V_{\text{cm,y}} = \frac{f_0 \sum_{i=1}^{N_{\text{max}}} n_{\text{p}}(\theta_i, \varphi_i)\sin(\theta_i + \varphi_i) - f_{\text{kin,y}}}{\gamma} \tag{4}$$

where $n_{\text{p}}(\theta_i, \varphi_i) = 1$ only if site i is occupied with a puller. As puller ants are not necessarily radially oriented, the system can develop torques. The angular speed is:

$$\omega = \frac{f_0 \sum_{i=1}^{N_{\text{max}}} n_{\text{p}}(\theta_i, \varphi_i)\sin(\varphi_i) - \tau_{\text{kin}}}{\gamma_{\text{rot}}} \tag{5}$$

Ants detach from and attach to the cargo. When attaching, the ants need to decide whether they will pull or lift. Detachment rates depend on the local velocity, as implied from Supplementary Fig. 7b. The detachment rate for the i'th ant is:

$$K^1_{\text{off}}\delta(f_{\text{loc}}) + K^2_{\text{off}}(1 - \delta(f_{\text{loc}})) \tag{6}$$

where $\delta(x) = 1, x > 0; \delta(x) = 0, x < 0$, $K_{\rm off}^1$ is the detachment rate from a non moving cargo and $K_{\rm off}^2$ is the detachment rate from a moving cargo. While attachment rate is $K_{\rm on}$ and is not affected by the velocity (see Supplementary Fig. 7c), the decision to be a puller or a lifter once attached depends on the local force. The probability to become a puller/lifer on site i is:

$$P_{\rm p} = \frac{1}{1 + \exp\left(-\frac{\mathbf{p}_i \cdot \mathbf{f}_{\rm loc}}{F_{\rm ind}}\right)}$$
$$P_{\rm l} = 1 - P_{\rm p} = \frac{1}{1 + \exp\left(\frac{\mathbf{p}_i \cdot \mathbf{f}_{\rm loc}}{F_{\rm ind}}\right)} \tag{7}$$

where $F_{\rm ind}$ is a measure for the individuality of the ant, $\mathbf{p}_i = (\cos(\theta_i), \sin(\theta_i))$ is the polarization vector of the i'th site. If $F_{\rm ind} \to \infty$ the ant decision is not affected by outside cues. If $F_{\rm ind} = 0$ the ant aligns with the motion she senses deterministically. If the ant decides to be a puller she aligns as closely as possible with the signal she receives. The angular range (i.e. φ) is limited to a window of angles: $[-\varphi_{\rm max}, +\varphi_{\rm max}]$. If the ant decides to be a lifter $\varphi_i = 0$ (she is aligned in the radial direction). $f_{\rm loc}$ is the signal at the attachment point and is composed from the translational forces (forces that act on the center of mass) and the torques. The ants try to oppose rotations and therefore given an attachment at point i, the ant senses the following force:

$$\mathbf{f}_{\rm loc}^i = \mathbf{f}_{\rm cm} - \mathbf{f}_{\rm rot}^i = \gamma \mathbf{V}_{\rm cm} + \frac{\gamma_{\rm rot}}{b} \mathbf{r}_i \times \boldsymbol{\omega} \tag{8}$$

where $\mathbf{V}_{\rm cm}$ is the center of mass velocity, $\boldsymbol{\omega}$ is the angular velocity and b is the outer radius of the object ($b = |\mathbf{r}_i|$). Notice that the sign of the rotational force is such that the ant oppose rotations.

Moreover, the ants can decide to switch between puller to lifter roles with a rate:

$$R_{\rm l \to p} = K_{\rm c} \exp\left(\frac{\mathbf{p}_i \cdot \mathbf{f}_{\rm loc}}{F_{\rm ind}}\right)$$
$$R_{\rm p \to l} = K_{\rm c} \exp\left(-\frac{\mathbf{p}_i \cdot \mathbf{f}_{\rm loc}}{F_{\rm ind}}\right) \tag{9}$$

Note that $K_{\rm c}$ denotes both the basal rate at which ants change their role when attached to a static cargo and the rate at which the pullers reorient themselves with the local velocity.

Supplementary Note 13 Parameter estimation

The parameters in the model are:

$$K_{\rm on}, K_{\rm off}^1, K_{\rm off}^2, K_{\rm c}, \gamma, \gamma_{\rm rot}, f_{\rm stat}, f_{\rm kin}, \tau_{\rm stat}, \tau_{\rm kin}, \beta, \Delta\varphi, F_{\rm ind}, N_{\rm av}, N_{\rm max}.$$

From Supplementary Fig. 7 b,c we estimate $K_{\rm off}^1 = 0.035\,{\rm sec}^{-1}$, $K_{\rm off}^2 = 0.01\,{\rm sec}^{-1}$ and $K_{\rm on} = 0.0017\,{\rm sec}^{-1}$. The rates of attachment and detachment are independent of the orientation with respect to the local force. Nevertheless, we see in Supplementary Fig. 7d that both in the model and in the clean board experiments the front is more occupied than the back. In the model, although the detachment/attachment rates are the same for the back and the

front, the front is more occupied as the front is defined with respect to the direction of the cargo's motion. Ants should exert forces at that direction and they are likely to be in the front. So from the definition of the front we see it must be more occupied than the back.

N_{av} is taken to be as in the clean board experiments, while N_{max} is estimated from the ratio of the object circumference to the width of an ant to be $N_{\mathrm{max}} = 20$.

The two parameters $\gamma, \gamma_{\mathrm{rot}}$ are not independent. We write the following equations:

$$V = \frac{f}{\gamma}$$
$$\omega = \frac{f}{\gamma_{\mathrm{rot}}} \tag{10}$$

Now we know that,

$$I\frac{d\omega}{dt} = bf \tag{11}$$

where b is the outer radius of the ring-like object and I is the moment of inertia, $I = \frac{1}{2}M(a^2 + b^2)$ (a is the inner radius of the ring). So we get $\gamma_{\mathrm{rot}} = \frac{1}{2}\frac{M}{\delta t}\frac{a^2+b^2}{b}$. Therefore,

$$\gamma_{\mathrm{rot}} = \frac{1}{2}\frac{a^2 + b^2}{b}\gamma \approx 0.4(cm)\gamma \tag{12}$$

where we plugged $a = 0.36cm, b = 0.57cm$ as the sizes of the object in the experiments. Therefore the signal felt by an ant in point i is (from equation (8)):,

$$\mathbf{f}^i_{\mathrm{loc}} = \gamma\left(\mathbf{v}_{\mathrm{cm}} + 0.7\mathbf{r}_i \times \boldsymbol{\omega}\right) \tag{13}$$

Simple mechanical measurements of the load on the board have yielded that $f_{\mathrm{kin}} \approx 0.9 f_{\mathrm{stat}}$ and that $f_{\mathrm{stat}} = 2.7 ant\,force$. From the standard deviation of the distribution in Supplementary Fig. 7e we estimate $\Delta\varphi = 52^o$. For a ring with $a = 0.36cm, b = 0.57cm$ the friction torque is $\tau_{\mathrm{stat/kin}} = 0.83 f_{\mathrm{stat/kin}}$.

We are left with 4 free parameters: $K_{\mathrm{c}}, F_{\mathrm{ind}}$ that are connected to the ants' decision making process and γ, β which have to do with the mechanics of the system. Simulating the model as described in the next Supplementary Note we find the free parameters that match the experimental data: $F_{\mathrm{ind}} = 10 ant force, K_{\mathrm{c}} = 0.7 sec^{-1}, \beta = 1.65, \gamma = 25 ant force \cdot sec \cdot cm^{-1}$. The results given these parameters are shown in Fig. 3 in the main text.

Supplementary Note 14 Gillespie code

In this Note we describe the algorithm step by step. As the model dynamics are stochastic we implement a stochastic simulation, namely Gillespie algorithm [3]. In each iteration we draw the time to the next event according to the total rate of all the possible reactions.

The first step in the algorithm is calculating the total rate. The total rate is the sum:

$$R_{\mathrm{tot}} = R_{\mathrm{att}} + R_{\mathrm{det}} + R_{\mathrm{con}} + R_{\mathrm{orient}} \tag{14}$$

This is a sum of the total rates of attachment, detachment, conversion and orientation events. The total attachment rate is simply K_{on} times the number of available ants times the number of empty sites:

$$R_{\mathrm{att}} = K_{\mathrm{on}} \cdot N_{\mathrm{av}} \cdot N_{empty} \tag{15}$$

The total detachment rate is the sum over all the occupied sites:

$$R_{\text{det}} = N_{\text{att}} \left(K_{\text{off}}^1 \delta(f_{\text{loc}}) + K_{\text{off}}^2 \left(1 - \delta(f_{\text{loc}})\right) \right) \tag{16}$$

where N_{att} is the number of attached ants and it was assumed that if $f_{\text{loc}} = 0$ for one site it is true for all sites. The total conversion rate is the sum,

$$R_{\text{con}} = K_{\text{c}} \sum_{i=1}^{N_{\text{max}}} \left(n_i^{\text{p}} \exp\left(-\frac{\mathbf{p}_i \cdot \mathbf{f}_{\text{loc}}^i}{F_{\text{ind}}} \right) + n_i^{\text{l}} \exp\left(\frac{\mathbf{p}_i \cdot \mathbf{f}_{\text{loc}}^i}{F_{\text{ind}}} \right) \right) \tag{17}$$

where n_i^{p} and n_i^{l} are the pullers and lifters occupancy operators. Finally, the rate of reorientation is given by

$$R_{\text{orient}} = K_{\text{c}} N^{\text{p}} \tag{18}$$

where N^{p} is the number of puller ants. Given the total rate we draw the time to the next event -*dt*- from an exponential distribution with mean $1/R_{\text{tot}}$. dt is generated by,

$$dt = -\frac{1}{R_{\text{tot}}} \log(rnd) \tag{19}$$

where rnd is drawn from a uniform distribution - $U(0,1)$. If the angular speed is zero: $\omega = 0$ then the center of mass of the cargo will move with velocity $\mathbf{v}_{\text{cm}}(t)$ so we update it by,

$$\begin{aligned} x_{\text{cm}}(t + dt) &= x_{\text{cm}}(t) + V_{\text{x}}(t)dt \\ y_{\text{cm}}(t + dt) &= y_{\text{cm}}(t) + V_{\text{y}}(t)dt \end{aligned} \tag{20}$$

If $\omega \neq 0$ the cargo rotates and the ants rotate with it. Therefore, their forces also rotate which cause the center of mass velocity to rotate as well. We divide the dt into many small time steps: $\delta t = 0.01 sec$. We update

$$x_{\text{cm}}(t + \delta t) = x_{\text{cm}}(t) + V_{\text{x}}(t)\delta t \tag{21}$$

$$y_{\text{cm}}(t + \delta t) = y_{\text{cm}}(t) + V_{\text{y}}(t)\delta t \tag{22}$$

$$\theta_i(t + \delta t) = \theta_i(t) + \omega \delta t \tag{23}$$

$$\mathbf{f}_i(t + \delta t) = \mathbf{f}_i(t) + \boldsymbol{\omega} \times \mathbf{f}_i(t)\delta t \tag{24}$$

$$\mathbf{v}_{\text{cm}}(t + \delta t) = \mathbf{v}_{\text{cm}}(t) + \boldsymbol{\omega} \times \mathbf{v}_{\text{cm}}(t)\delta t \tag{25}$$

until a time interval of dt has passed.

The next step after determining dt is to determine what is going to happen. For that we generate another random number -*rnd*- from a uniform distribution $U(0,1)$.

If $rnd < R_{\text{att}}/R_{\text{tot}}$ an attachment event occurs. Each empty site has the same probability to be occupied. Once a site is chosen the decision to be a puller or a lifter is made with respect to $\mathbf{f}_{\text{loc}}^i(t)$ and $\mathbf{p}_i(t)$ in equation (7)).

If $R_{\text{att}}/R_{\text{tot}} \leq rnd < (R_{\text{att}} + R_{\text{det}})/R_{\text{tot}}$ a detachment event occurs. Each ant has an equal probability to detach.

If $(R_{\text{att}} + R_{\text{det}})/R_{\text{tot}} \leq rnd < (R_{\text{att}} + R_{\text{det}} + R_{\text{con}})/R_{\text{tot}}$ a conversion event occurs. The conversion events are not equally probable. We choose the event by its weight with respect to $\mathbf{f}_{\text{loc}}(t)$ and $\mathbf{p}_i(t)$ in equation (9).

If $rnd \geq (R_{\text{att}} + R_{\text{det}} + R_{\text{con}})/R_{\text{tot}}$ we randomly choose a puller so she aligns with the local velocity.

The last stage is to calculate the velocity $\mathbf{v}_{\text{cm}}(t+dt)$ by equation (4) and angular velocity by equation (5).

Supplementary Note 15 Continuous forgetting process and the case of non-uniform F_{ind}'s

In the main text Fig. 4a an informed ant loses it's memory in a sharp way. Either it is informed or not and there is no gradual forgetting process, but rather a stochastic process of irreversible forgetting. We now want to explore what is the effect of modelling this forgetting process such that it is gradual. We therefore implement the same procedure as in Fig. 4a (main text), but with an informed ant that loses its information continuously. In the simulation this is achieved by allowing the informed ant to be, in each point in time, in one of two states, either it pulls in the $x > 0$ direction (to the nest) or it behaves as the rest of the uninformed ants. The rate of switching between these two states decays exponentially with a time constant $t_{\text{forg}} = 10\,sec$. The rates for this switching process (following the time of attachment, $t = 0$) are taken to be: $k_{\text{on}}^{\text{informed}} = K_{\text{c}} \exp\left(-t/2t_{\text{forg}}\right), k_{\text{off}}^{\text{informed}} = K_{\text{c}} \exp\left(t/2t_{\text{forg}}\right)$, $K_c = 0.7\,sec^{-1}$. The attached informed ant will be mostly in the informed state just after the attachment, and mostly uninformed after $10\,sec$, but the decay is gradual. We show the response for such an ant in Supplementary Fig. 13. Compared with main text Fig. 4a we see that the response of the ants is still close to the maximum, although the response at large F_{ind} (the unimodal phase) saturates to a higher value.

We also check what happens if the F_{ind} value is taken from a distribution. That is, the individuality parameter is different among different ants and realizations of the simulation. As $F_{\text{ind}} > 0$ we choose F_{ind} to be drawn from a log-normal distribution. We generate a random variable by,

$$F_{\text{ind}} = e^{\mu+\sigma z} \tag{26}$$

where z is a normal random variable with a mean 0 and std 1. The mean is given by,

$$\langle F_{\text{ind}} \rangle = e^{\mu+\frac{\sigma^2}{2}} \tag{27}$$

and variance,

$$\text{Var}\left(F_{\text{ind}}\right) = \left(e^{\sigma^2} - 1\right) e^{2\mu+\sigma^2} \tag{28}$$

We choose the two parameters μ, σ to be $\mu = 2.2226, \sigma = 0.4$ such that,

$$\langle F_{\text{ind}} \rangle = 10 \tag{29}$$

and variance,

$$\text{Var}\left(F_{\text{ind}}\right) = 17.3516 \tag{30}$$

We show the pdf of the distribution in Supplementary Fig. 8a. The simulation results agree with the experimental data in Supplementary Fig. 8b-d. The response of a system with a distribution of F_{ind} to an informed ant (that forgets the information gradually) is shown in Supplementary Fig. 9. We see that the response is close to the maximal value and qualitatively the curve looks like the one of main text Fig. 4a. Yet, again the behavior at the unimodal phase (right side) saturate to a higher value. We find the results hold in a regime where the variance is not too big (at $\text{Var}\left(F_{\text{ind}}\right) \approx 30$ the system behavior is too persistent).

Supplementary Note 16 Uninformed load motion

Load motion in experiments on a clean board, with no freely moving ants on the board, follows a random walk pattern $\langle r \rangle \sim \sqrt{t}$ (Supplementary Fig. 10). Note that during the earlier parts of the carrying there are more stops and less carrying ants, explaining why the experimental results show a slower initial rise than evident in the fit.

Supplementary Note 17 Speed distribution constraints

Unlike natural, non-manipulated cooperative transport, group food retrieval on a clean board, with no scent markers and knowledgeable ants, is characterized by stops of various durations. Continuous periods where $v < 0.02$ were defined as stops. The distribution of stop durations on a log scale is bimodal (Supplementary Fig. 11a). This motivates an exclusion criterion based on the duration, since long duration stops are simply not part of the carrying. Thus, the distribution of speeds shown in main text Fig. 3b is constrained to include only short stops ($T < 5.25sec$). Also neglected were short periods of carrying between stops ($T < 2sec$), eliminating image processing artifacts. The distribution was also constrained to include only frames where the number of carrying ants was larger than four; this in order to make doubly sure only frames where actual carrying took place are included in the data. The full distribution, including all stops (but still constrained to $N > 4$), is shown in Supplementary Fig. 11b.

Supplementary Note 18 Cosine correlation function calculation

In Fig. 3c of the main text, the cosine correlation functions of the experimental and simulated trajectories are compared. To calculate these, the following procedure was performed: First, for each trajectory a spline function was fit and divided it into equal parts 0.12 cm long. From these parts, "velocities" were defined as the difference between consecutive equidistant points, namely, $\mathbf{v}_i = \mathbf{x}_{i+1} - \mathbf{x}_i$. The directions of these velocity vectors θ_i were used to calculate the correlation function. For each pair of equidistant points i, j, the difference in direction was quantified using $\cos(\theta_i - \theta_j)$. Averaging the results of this calculation for all pairs of points for every distance separately gives us the cosine correlation function of the trajectories.

Supplementary Note 19 Varying K_c and F_{ind}

We show results for other K_{c} values in Supplementary Fig. 12a,b. K_{c} is the rate for two processes: reorientation and conversion between roles. Model fits to the speed, angular speed and cosine correlations for different values of K_{c} are presented in Supplementary Fig. 12a-b. We note that when the conversion rate is large the motion is more persistent.

The effects of varying the parameter F_{ind} are presented in Supplementary Fig. 12c,d. The smaller the value of F_{ind} the more the ants are sensitive to the local motion in their decision of whether to be a puller or a lifter. For $F_{\mathrm{ind}} \to 0$ the ants within $[-\pi/2, \pi/2]$ from the velocity direction (the leading edge) are pullers and outside this range they are lifters. This leads to more persistent motions. For $F_{\mathrm{ind}} \to \infty$ the decision for being a puller or a

lifter is unbiased and this leads to a random walk collective motion with a short persistent length and smaller speed. This is indeed observed in Supplementary Fig. 12c,d.

Supplementary Note 20 Code for informed ants

An ant with information has the same parameters as the other ants but she orients herself toward the positive x direction (and not with respect to the sensed forces as the other ants). Motivated by Figs. 2a and 2c in the main text a knowledgeable ant forgets her knowledge with a rate $K_{\text{forget}} = 0.1sec^{-1}$. We examine the response for informed ants using two approaches: the first corresponds to the main plot in Fig. 4a in the main text. In this scheme a single informed ant is attached to a cargo in the setting of the clean board. We take the average number of surrounding ants in clean board experiments ($N_{\text{av}} = 11$), and measure the movement along the x direction in a time interval which is defined as the average time between two successive attachments. In Fig. 4 in the main text we show the results of this approach after averaging over many realizations ($\sim 8 \cdot 10^4$) and smoothing the curve with the Matlab function 'smooth'.

In Fig. 4 in the main text we see that the response for $F_{\text{ind}} \to \infty$ is $\langle x \rangle \approx 0.49cm$. As the mean time between attachments ($\approx 14sec$) is greater than the mean time of informed ant forgetting her information ($= 10sec$), the main contribution is from the first 10 seconds. As we assume that lifters reduce the friction and the other pullers' forces average to zero ($F_{\text{ind}} \to \infty$) we get:

$$\langle x \rangle_{main} = \frac{f}{\gamma \cdot K_{\text{forget}}} = \frac{10}{25} = 0.4cm \tag{31}$$

Where $\frac{1}{K_{\text{forget}}} = 10sec$ is the disorientation timescale of informed ants as specified in the main text. We need to add the average distance passed after the informed ant forgets the knowledge. This is the time until either a reorientation or conversion events. We get

$$\langle x \rangle = 0.4cm + \frac{f}{\gamma} \cdot \frac{1}{K_{\text{c}}} = 0.4571cm \tag{32}$$

which is quite close to the simulated result of $0.49cm$. Our estimation is lower than the actual result as we assumed there is no x-component after one event but the ants that reoriented to help the informed ant might still be orienting toward the nest.

The second approach is to test the response for longer times as shown in the inset of Fig. 4a in the main text. In this scheme we apply a constant influx of knowledgeable ants ($K_{\text{know}} = 0.03\,\text{sec}^{-1}$) on top of the clean board conditions such that ~ 1.2 informed ants are attached on average which is close to the experimental estimation.

Supplementary Note 21 Scaling in the detailed model

In this Note we describe how scaling affects the effective conformity of the ants. Bigger systems are more persistent and are controlled by an effectively smaller F_{ind}. To see this consider a bigger cargo, it is characterized by a bigger circumference and therefore bigger N_{max}. For simplicity consider a case where the friction is zero due to lifters. We then get

the following average:

$$\mathbf{f}_{\mathrm{m}} = \sum_{i}^{N_{\max}} \frac{\mathbf{f}_i}{N_{\max}} \tag{33}$$

where f_i is non zero only for sites occupied with pullers and f_{m} is the average force.

The occupation of a site by a puller in our model is a function of: $\mathbf{p}_i \cdot \sum_i \mathbf{f}_i / F_{\mathrm{ind}}$, which can also be written as: $\mathbf{p}_i \cdot N_{\max}\mathbf{f}_{\mathrm{m}}/F_{\mathrm{ind}}$. It is therefore equivalent to the original system but with a rescaled individuality parameter: $\tilde{F}_{\mathrm{ind}} = F_{\mathrm{ind}}/N_{\max}$.

As we increase $N_{\max}$, $\tilde{F}_{\mathrm{ind}}$ is reduced and f_{m} has a bigger value (see also equation (41) in Supplementary Note 22 for a scaling relation in a 1D system). Moreover simulation results imply that a phase transition occur where f_{m} goes from zero to a non zero value in a continuous manner (as seen from velocity component distribution, main text Figs. 3b and 4a).

We also wish to examine the responses for cargoes of different sizes. We consider the cargo as a zero-thickness ring with a radius b, given that the radius grows/shrinks by a factor S we get:

$$\begin{aligned}
b' &= S \cdot b \\
N'_{\max} &= S \cdot N_{\max} \\
N'_{\mathrm{av}} &= S \cdot N_{\mathrm{av}} \\
K'_{\mathrm{know}} &= S \cdot K_{\mathrm{know}} \\
f'_{\mathrm{stat}} &= S \cdot f_{\mathrm{stat}} \\
\gamma' &= S \cdot \gamma \\
\gamma'_{\mathrm{rot}} &= b' \cdot \gamma' = S^2 \gamma_{\mathrm{rot}}
\end{aligned} \tag{34}$$

We then examine the deviation from optimality for this scheme in Fig. 4b in the main text where we measure the displacement in the nest direction after the time between successive attachments has passed and average over many realizations ($10^4 - 10^5$) and smooth the results with the Matlab function 'smooth'.

In Supplementary Fig. 17 we compare the translational and angular speed distributions of normal and large sized objects. The radius of the large object is bigger by a factor of 7 and the mass (which affects γ and friction) is bigger by a factor of 8.2. Furthermore, we take the geometry to be as in the experiments where the normal sized object is a thick ring while the heavy object is a thin ring. Indeed, as observed in the experiments (see Supplementary Fig. 17), we find that larger objects tend to move faster and with less rotations.

Supplementary Note 22 Fully connected Ising model for motion in 1D

Consider N ants carrying an object in 1D, such that $N/2$ ants are on one edge and the other $N/2$ on the opposite edge. In this setting, pullers on one side exert a force to $x > 0$ direction while at the other side they exert a force to the $x < 0$ direction. Illustration of the model is shown in Supplementary Fig. 14a. The $x > 0$ direction is referred as the front while the $x < 0$ direction is the back. Defining σ_a as,

$$\sigma_a = \begin{cases} -1; & \textit{lifter} \\ 1; & \textit{puller} \end{cases} \tag{35}$$

Where a indicates whether the ant is in the front ($a = 1$) or in the back ($a = -1$). If the pullers distributed randomly between back and front the cargo will not move anywhere (it will perform a random walk). The sum over all pullers is zero. However, the decision (to become a puller/lifter) of the ant depends on whether she's in the back/front and the total force. We implement it in the following way:
The rate of a puller becoming a lifter is $r_{\mathrm{p}\to\mathrm{l}} = \exp\left(-\frac{af_{\mathrm{tot}}}{F_{\mathrm{ind}}}\right)$;
The rate of a lifter becoming a puller is $r_{\mathrm{l}\to\mathrm{p}} = \exp\left(+\frac{af_{\mathrm{tot}}}{F_{\mathrm{ind}}}\right)$.
where F_{ind} is the individuality parameter (which sets the sensitivity of the ant's decision rule to the local forces) and $a = +1$ ($= -1$) for front (back). We see from equation (35) that the occupancy operator of pullers (lifters) is $n^a_{\mathrm{p(l)}} = \left(+(-)\sigma_a + 1\right)/2$ and therefore the total force is

$$f_{\mathrm{tot}} = f \sum_{front} \frac{\sigma_1 + 1}{2} - f \sum_{back} \frac{\sigma_{-1} + 1}{2} \tag{36}$$

In steady state the rates from and into a puller state should be equal in the front and in the back:

$$n^a_{\mathrm{p}} \exp\left(-\frac{af_{\mathrm{tot}}}{F_{\mathrm{ind}}}\right) = n^a_{\mathrm{l}} \exp\left(+\frac{af_{\mathrm{tot}}}{F_{\mathrm{ind}}}\right) \tag{37}$$

Therefore, at steady state, the mean number of pullers in the leading edge is:

$$\frac{\langle\sigma_i\rangle + 1}{2} = \left\langle \frac{\exp\left(+\frac{f_{\mathrm{tot}}}{F_{\mathrm{ind}}}\right)}{2\cosh\left(\frac{f_{\mathrm{tot}}}{F_{\mathrm{ind}}}\right)} \right\rangle \tag{38}$$

while in the back it is:

$$\frac{\langle\sigma_i\rangle + 1}{2} = \left\langle \frac{\exp\left(-\frac{f_{\mathrm{tot}}}{F_{\mathrm{ind}}}\right)}{2\cosh\left(\frac{f_{\mathrm{tot}}}{F_{\mathrm{ind}}}\right)} \right\rangle \tag{39}$$

As f_{tot} is a sum of N random variables we can assume it's fluctuations are small. This is a mean field assumption and it is valid for our system as all the ants interact with each other through the cargo. Therefore, the force every ant feels (the effective field she feels) is composed from N contributions and the dynamics (and steady state) are mean field in nature. Since the fluctuations are small we can write,

$$\left\langle \frac{\exp\left(+\frac{f_{\mathrm{tot}}}{F_{\mathrm{ind}}}\right)}{2\cosh\left(\frac{f_{\mathrm{tot}}}{F_{\mathrm{ind}}}\right)} \right\rangle = \frac{\exp\left(+\frac{\langle f_{\mathrm{tot}}\rangle}{F_{\mathrm{ind}}}\right)}{2\cosh\left(\frac{\langle f_{\mathrm{tot}}\rangle}{F_{\mathrm{ind}}}\right)} \tag{40}$$

and get a closed self consistent equation for $\langle f_{\mathrm{tot}}\rangle$ which we call now simply f_{tot}.

Writing the self consistent equation for f_{tot} we get:

$$f_{\mathrm{tot}} = f\frac{N}{2}\tanh\left(\frac{f_{\mathrm{tot}}}{F_{\mathrm{ind}}}\right) \tag{41}$$

defining $F_{\rm ind}^{\rm c} = f\frac{N}{2}$ and assuming the $f_{\rm tot}$ is continuous at the transition (2nd order phase transition), we can approximate for small $f_{\rm tot}$.

$$f_{\rm tot} = F_{\rm ind}^{\rm c}\left(\frac{f_{\rm tot}}{F_{\rm ind}} - \frac{f_{\rm tot}^3}{3F_{\rm ind}^3}\right) \tag{42}$$

A non zero solution exists when:

$$F_{\rm ind} < F_{\rm ind}^{\rm c} = N\frac{f}{2} \tag{43}$$

Which demonstrates that the critical value of $F_{\rm ind}$ linearly depends on system size. This implies that if $F_{\rm ind}$, an internal decision making parameter of the ant, remains constant as system size varies then larger systems must be more ordered.

Calculating $f_{\rm tot}$ and the susceptibility when $F_{\rm ind}$ is close to $F_{\rm ind}^{\rm c}$ we get,

$$f_{\rm tot} = \pm\sqrt{3}F_{\rm ind}^{\rm c}\sqrt{\frac{F_{\rm ind}^{\rm c} - F_{\rm ind}}{F_{\rm ind}^{\rm c}}} \tag{44}$$

and for the susceptibility, χ, we get:

$$\chi = \begin{cases} \frac{F_{\rm ind}^{\rm c}}{F_{\rm ind} - F_{\rm ind}^{\rm c}}; & F_{\rm ind} > F_{\rm ind}^{\rm c} \\ \frac{F_{\rm ind}^{\rm c}}{2(F_{\rm ind}^{\rm c} - F_{\rm ind})}; & F_{\rm ind} < F_{\rm ind}^{\rm c} \end{cases} \tag{45}$$

Our results give the expected exponents of Landau mean field theory: $\beta = 1/2, \gamma = \gamma' = 1$.

Supplementary Note 23 1D response for fully connected Ising model for short times

Assume that the nest is in the positive direction and that at $t = 0$ an ant at the leading edge becomes informed. This ant knows that the positive direction is the correct direction and will therefore persist as a puller for some extended period of time. We aim to estimate the short time scale response of the system to such an event. Consider a short time scale δt so small that at most one event can occur. First, consider $F_{\rm ind} \to \infty$ such that there is no response. In that case we need to sum the $N/2 - 1$ sites available at the front (one site is occupied by the informed ant) where at each site there is *probability* $= 1/2$ to have a puller; then we need to subtract the sum of $N/2$ forces of the back part where like in the front a site is occupied with a puller with *probability* $= 1/2$:

$$f_{\rm tot}(\delta t) = f\left(\frac{N}{2} - 1\right)\frac{1}{2} - f\frac{N}{2}\frac{1}{2} + f = \frac{f}{2} \tag{46}$$

This is the total force when the system is not responsive. We now turn to consider finite values of the parameter $F_{\rm ind}$. We begin with the paramagnetic phase ($F_{\rm ind} > F_{\rm ind}^{\rm c}$) where $f_{\rm tot} = 0$ before $t = 0$. At $t = 0$, $f_{\rm tot}(t = 0) = f/2$ as the system did not response yet.

The force difference $\Delta f_{\rm tot}(\delta t)$ is a random variable with probability:

$$\Delta f_{\rm tot}(\delta t) = \begin{cases} +f; & probability = p_+ \\ -f; & probability = p_- \end{cases} \tag{47}$$

where,

$$p_+ = (rate \;\; lifter \rightarrow puller, \;\; front \quad + \quad rate \;\; puller \rightarrow lifter, \;\; back)\, \delta t \tag{48}$$
$$p_- = (rate \;\; puller \rightarrow lifter, \;\; front \quad + \quad rate \;\; lifter \rightarrow puller, \;\; back)\, \delta t \tag{49}$$

Using the approximation $exp\left(\pm\frac{f_{\rm tot}}{F_{\rm ind}}\right) \approx 1 \pm \frac{f_{\rm tot}}{F_{\rm ind}}$ as the force is small and remembering that the occupation of pullers/lifter is still 1/2 (since no event occurred yet) we get

$$p_+ = \left(\frac{N}{2} - 1\right)\frac{1}{2}\left(1 + \frac{f_{\rm tot}(0)}{F_{\rm ind}}\right)\delta t + \left(\frac{N}{2}\right)\frac{1}{2}\left(1 + \frac{f_{\rm tot}(0)}{F_{\rm ind}}\right)\delta t \tag{50}$$
$$p_- = \left(\frac{N}{2} - 1\right)\frac{1}{2}\left(1 - \frac{f_{\rm tot}(0)}{F_{\rm ind}}\right)\delta t + \left(\frac{N}{2}\right)\frac{1}{2}\left(1 - \frac{f_{\rm tot}(0)}{F_{\rm ind}}\right)\delta t \tag{51}$$

Taking the average of $\Delta f_{\rm tot}(\delta t)$ we get,

$$\Delta f_{\rm tot}(\delta t) = f(p_+ - p_-) = N\frac{f_{\rm tot}(0)}{F_{\rm ind}}\delta t \tag{52}$$

Plugging $f_{\rm tot}(0) = f/2$ we get for $F_{\rm ind} > F^{\rm c}_{\rm ind}$:

$$\Delta f_{\rm tot}(\delta t) = \frac{f^2}{2}\frac{N}{F_{\rm ind}}\delta t \tag{53}$$

We now turn to the ferromagnetic phase $F_{\rm ind} < F^{\rm c}_{\rm ind}$. In this phase $f_{\rm tot} \neq 0$ and we need to average over both directions: positive and negative. Each of the directions has probability 1/2. When the cargo moves in the positive direction the force just after an ant at the leading edge becomes informed is:

$$f_{\rm tot}(0^+) = f\left(\frac{N}{2} - 1\right)\frac{\exp\left(\frac{f_{\rm tot}}{F_{\rm ind}}\right)}{2\cosh\left(\frac{f_{\rm tot}}{F_{\rm ind}}\right)} - f\frac{N}{2}\frac{\exp\left(-\frac{f_{\rm tot}}{F_{\rm ind}}\right)}{2\cosh\left(\frac{f_{\rm tot}}{F_{\rm ind}}\right)} + f \tag{54}$$
$$= |f_{\rm tot}| + f\frac{\exp\left(-\frac{f_{\rm tot}}{F_{\rm ind}}\right)}{2\cosh\left(\frac{f_{\rm tot}}{F_{\rm ind}}\right)} \tag{55}$$

As the cargo moves in the positive direction there is more probability that an ant in the front is a puller before she became informed. Therefore, the informed ant contribution is smaller than $f/2$. If the cargo moves in the negative direction:

$$f_{\rm tot}(0^+) = -|f_{\rm tot}| + f\frac{\exp\left(\frac{f_{\rm tot}}{F_{\rm ind}}\right)}{2\cosh\left(\frac{f_{\rm tot}}{F_{\rm ind}}\right)} \tag{56}$$

As the cargo moves in the minus direction there is more probability that an ant in the front is a lifter before she became informed. Therefore, the informed ant contribution is bigger than $f/2$.

The force at time δt is

$$f_{\text{tot}}(\delta t) = \left(|f_{\text{tot}}| + f \frac{\exp\left(-\frac{f_{\text{tot}}}{F_{\text{ind}}}\right)}{2\cosh\left(\frac{f_{\text{tot}}}{F_{\text{ind}}}\right)} + \Delta f^{+}_{\text{tot}} \right) \frac{1}{2} + \left(-|f_{\text{tot}}| + f \frac{\exp\left(\frac{f_{\text{tot}}}{F_{\text{ind}}}\right)}{2\cosh\left(\frac{f_{\text{tot}}}{F_{\text{ind}}}\right)} + \Delta f^{-}_{\text{tot}} \right) \frac{1}{2} \tag{57}$$

$$= \frac{f}{2} + \frac{\Delta f^{+}_{\text{tot}}(\delta t) + \Delta f^{-}_{\text{tot}}(\delta t)}{2} \tag{58}$$

Now as $f_{\text{tot}} \neq 0$ we need to take into account the exponential form of the puller/lifter probability and of the rates.

$$p_{+} = \left(\frac{N}{2} - 1\right) \frac{\exp\left(-\frac{f_{\text{tot}}(0)}{F_{\text{ind}}}\right)}{2\cosh\left(\frac{f_{\text{tot}}(0)}{F_{\text{ind}}}\right)} \exp\left(\frac{f_{\text{tot}}(0) + f \frac{\exp\left(-\frac{f_{\text{tot}}}{F_{\text{ind}}}\right)}{2\cosh\left(\frac{f_{\text{tot}}}{F_{\text{ind}}}\right)}}{F_{\text{ind}}} \right) \delta t + \tag{59}$$

$$\left(\frac{N}{2}\right) \frac{\exp\left(\frac{f_{\text{tot}}(0)}{F_{\text{ind}}}\right)}{2\cosh\left(\frac{f_{\text{tot}}(0)}{F_{\text{ind}}}\right)} \exp\left(-\frac{f_{\text{tot}}(0) + f \frac{\exp\left(-\frac{f_{\text{tot}}}{F_{\text{ind}}}\right)}{2\cosh\left(\frac{f_{\text{tot}}}{F_{\text{ind}}}\right)}}{F_{\text{ind}}} \right) \delta t \tag{60}$$

$$\approx \frac{N}{2} \frac{1 + \frac{f}{F_{\text{ind}}} \frac{\exp\left(-\frac{f_{\text{tot}}}{F_{\text{ind}}}\right)}{2\cosh\left(\frac{f_{\text{tot}}}{F_{\text{ind}}}\right)}}{\cosh\left(\frac{f_{\text{tot}}(0)}{F_{\text{ind}}}\right)} \delta t \tag{61}$$

Similarly,

$$p_{-} \approx \frac{N}{2} \frac{1 - \frac{f}{F_{\text{ind}}} \frac{\exp\left(-\frac{f_{\text{tot}}}{F_{\text{ind}}}\right)}{2\cosh\left(\frac{f_{\text{tot}}}{F_{\text{ind}}}\right)}}{\cosh\left(\frac{f_{\text{tot}}(0)}{F_{\text{ind}}}\right)} \delta t \tag{62}$$

The force difference in the positive direction regime is

$$\Delta f^{+}_{\text{tot}}(\delta t) = \frac{f^2}{2} \frac{N \exp\left(-\frac{f_{\text{tot}}(0)}{F_{\text{ind}}}\right)}{F \cosh^2\left(f_{\text{tot}}(0)/F_{\text{ind}}\right)} \delta t \tag{63}$$

If the cargo moves in the negative direction we get:

$$\Delta f^{-}_{\text{tot}}(\delta t) = \frac{f^2}{2} \frac{N \exp\left(+\frac{f_{\text{tot}}(0)}{F_{\text{ind}}}\right)}{F \cosh^2\left(f_{\text{tot}}(0)/F_{\text{ind}}\right)} \delta t \tag{64}$$

Summing the two contributions we get the full answer:

$$\Delta f_{\text{tot}}(\delta t) = \begin{cases} \frac{f^2}{2} \frac{N}{F_{\text{ind}}} \delta t; & F_{\text{ind}} > F^{\text{c}}_{\text{ind}} \\ \frac{f^2}{2} \frac{N}{F_{\text{ind}}} \frac{1}{\cosh\left(\frac{f_{\text{tot}}(0)}{F_{\text{ind}}}\right)} \delta t; & F_{\text{ind}} < F^{\text{c}}_{\text{ind}} \end{cases} \tag{65}$$

The maximum is at $F_{\text{ind}} = F_{\text{ind}}^{\text{c}}$ but it is not diverging (unlike the susceptibility). If we divide both sides by δt we see we have a derivative wrt time at $t = 0$, while susceptibility is derivative wrt magnetic field at long times. It's easier to plot when we normalize the result by the maximum:

$$\frac{\delta f_{\text{tot}}(0)}{\delta t} = \begin{cases} f\frac{F_{\text{ind}}^{\text{c}}}{F_{\text{ind}}}; & F_{\text{ind}} > F_{\text{ind}}^{\text{c}} \\ f\frac{F_{\text{ind}}^{\text{c}}}{F_{\text{ind}}}\frac{1}{\cosh\left(\frac{f_{\text{tot}}(0)}{F_{\text{ind}}}\right)}; & F_{\text{ind}} < F_{\text{ind}}^{\text{c}} \end{cases} \tag{66}$$

We plot this result in Fig. 4c in the main text.

Now examine equation (41), we can write it as

$$f_{\text{tot}} = F_{\text{ind}}^{\text{c}} \tanh\left(\frac{f_{\text{tot}}}{F_{\text{ind}}}\right) \tag{67}$$

where $F_{\text{ind}}^{\text{c}} = Nf/2 = Nf_{\text{ind}}^{\text{c}}$. So increasing N keeping F_{ind} constant is like increasing $F_{\text{ind}}^{\text{c}}$ and f_{tot} is a function of N. We have,

$$\frac{\delta f_{\text{tot}}(0)}{\delta t} = \begin{cases} \frac{f^2}{2F_{\text{ind}}}N; & N < F_{\text{ind}}/f_{\text{c}} \\ \frac{f^2}{2F_{\text{ind}}}\frac{N}{\cosh\left(\frac{f_{\text{tot}}(t=0,N)}{F_{\text{ind}}}\right)}; & N > F_{\text{ind}}/f_{\text{c}} \end{cases} \tag{68}$$

We plot this result in Fig. 4d in the main text. We note that by taking the mean number of attached ants from the clean board simulations plus an informed ant ($N \approx 8.6$) and using $F_{\text{ind}}^{\text{c}} = Nf/2$ we get $F_{\text{ind}}^{\text{c}} \approx 4.3$ which is quite close to the maximum in Fig. 4a in the main text (≈ 4.2).

Supplementary Note 24 Order parameter of the mesoscopic order-disorder transition.

Given the analysis of the last two Supplementary Notes we set to check whether we see the order-disorder transition in the finite system simulation. The calculation was carried under a mean field assumption, i.e. $\langle g(f_{\text{tot}})\rangle = g\left(\langle f_{\text{tot}}\rangle\right)$ yet this is not true for a finite size system with non zero fluctuations. The most obvious discrepancy is that in the mean field limit the speed above the transition F_{ind} is zero while in a finite system the speed distribution has a non-zero width and therefore the mean speed is not zero. We therefore compare the results of the mean field system with a finite size system.

We begin the analysis by considering a finite 1D system with attachments and detachments with rates and size as defined in Supplementary Note 13. The average number of attached ants is 7.6 (as in the clean board experiment). Therefore, the mean-field solution (equation (43)) F_{ind} of the transition is expected to be close to $F_{\text{ind}}^{\text{c}} = 3.8$. In Supplementary Fig. 15 we plot two quantities that can serve as an order parameter as a function of F_{ind}. The first is the mean speed,

$$V = \frac{n_1^{\text{p}} - n_{-1}^{\text{p}} - \left(f_k - \beta n^{\text{l}}\right)\theta\left(f_k - \beta n^{\text{l}}\right)}{\gamma} \tag{69}$$

where $1, -1$ denote front, back respectively and γ and β are taken to be as in Supplementary Note 13, f_k is the kinetic friction and $\theta(x)$ is the Heaviside function. The second quantity that could serve as an order parameter is the mean difference between pullers and lifters or the surplus defined as,

$$S = \sum_{a=-1,1} \frac{\left(n_a^{\mathrm{p}} - n_a^{\mathrm{l}}\right) a \cdot V}{N_{\max}|V|} \tag{70}$$

We see in Supplementary Fig. 15 that although the transition is not sharp as in the mean-field computation (equation (41)), the critical mean-field value $F_{\mathrm{ind}}^{\mathrm{c}} = 3.8$ is close to the inflection point of the curve for the 1D mesoscopic model.

We proceed to compare the results for the 2D system as described in Supplementary Notes 12-13 and in Supplementary Fig. 16a. We indeed find that an increase in both the mean speed and the surplus is occurring near the same critical value found in the 1D mean-field calculation $F_{\mathrm{ind}}^{\mathrm{c}}$ (as in Supplementary Fig. 15). We note the increase is more pronounced in the surplus curve. We also plot the order parameters as a function of the scale in Supplementary Fig. 16b. By comparing the results of the 2D system with the results of the 1D system we see that the curves for the order parameters look similar. This similarity supports our use of the 1D model (studied in Supplementary Notes 22-23) to shed light on the transition we see in simulating the full 2D system. The underlying reason is that the ants act to orient against rotations, which means that rotational motion can not contribute to the ordering transition, which is driven only by the linear motion of the load. This point is evident when we cancel rotations in the system (simply setting the angular speed to zero, no matter what torques are acting on the cargo) and we see in Supplementary Fig. 16a that both order parameters look almost the same. Therefore, the 1D model describes the transition of the 2D model very well. The similarity is further enhanced by the fact that the ants can reorient towards the direction of motion, such that they are not radially oriented around the circular load, and thereby become overall more 1D-like.

Supplementary Note 25 Large object motion

Collective transport of large rings ($4cm$ in radius) was characterized by persistent smooth motion. The resulting trajectories are straight, have very little detours and possess no loops (see Supplementary Fig. 17, main text Fig. 4e).

Supplementary References

[1] Trager, J. C. A revision of the genus paratrechina of the continental united states. *Sociobiology* **9**, 51–162 (1984).

[2] Robson, S. K. & Traniello, J. F. Transient division of labor and behavioral specialization in the ant formica schaufussi. *Naturwissenschaften* **89**, 128–131 (2002).

[3] Gillespie, D. Exact stochastic simulation of coupled chemical reactions. *J. Phys. Chem.* **81**, 2340–2361 (1977).
